# Machine learning-based identification of an immunotherapy-related signature to enhance outcomes and immunotherapy responses in melanoma

**DOI:** 10.3389/fimmu.2024.1451103

**Published:** 2024-09-17

**Authors:** Zaidong Deng, Jie Liu, Yanxun V. Yu, Youngnam N. Jin

**Affiliations:** ^1^ Department of Neurology, Medical Research Institute, Zhongnan Hospital of Wuhan University, Wuhan University, Wuhan, China; ^2^ Frontier Science Center for Immunology and Metabolism, Wuhan University, Wuhan, China

**Keywords:** skin cutaneous melanoma, immunotherapy, tumor immune microenvironment (TIME), machine learning, multi-omics, GBP5, biomarker

## Abstract

**Background:**

Immunotherapy has revolutionized skin cutaneous melanoma treatment, but response variability due to tumor heterogeneity necessitates robust biomarkers for predicting immunotherapy response.

**Methods:**

We used weighted gene co-expression network analysis (WGCNA), consensus clustering, and 10 machine learning algorithms to develop the immunotherapy-related gene model (ITRGM) signature. Multi-omics analyses included bulk and single-cell RNA sequencing of melanoma patients, mouse bulk RNA sequencing, and pathology sections of melanoma patients.

**Results:**

We identified 66 consensus immunotherapy prognostic genes (CITPGs) using WGCNA and differentially expressed genes (DEGs) from two melanoma cohorts. The CITPG-high group showed better prognosis and enriched immune activities. DEGs between CITPG-high and CITPG-low groups in the TCGA-SKCM cohort were analyzed in three additional melanoma cohorts using univariate Cox regression, resulting in 44 consensus genes. Using 101 machine learning algorithm combinations, we constructed the ITRGM signature based on seven model genes. The ITRGM outperformed 37 published signatures in predicting immunotherapy prognosis across the training cohort, three testing cohorts, and a meta-cohort. It effectively stratified patients into high-risk or low-risk groups for immunotherapy response. The low-risk group, with high levels of model genes, correlated with increased immune characteristics such as tumor mutation burden and immune cell infiltration, indicating immune-hot tumors with a better prognosis. The ITRGM’s relationship with the tumor immune microenvironment was further validated in our experiments using pathology sections with GBP5, an important model gene, and CD8 IHC analysis. The ITRGM also predicted better immunotherapy response in eight cohorts, including urothelial carcinoma and stomach adenocarcinoma, indicating broad applicability.

**Conclusions:**

The ITRGM signature is a stable and robust predictor for stratifying melanoma patients into ‘immune-hot’ and ‘immune-cold’ tumors, enhancing prognosis and response to immunotherapy.

## 1 Introduction

Skin cutaneous melanoma (SKCM), the most aggressive skin cancer, exhibits high heterogeneity and a poor prognosis. Over the past few decades, the incidence of SKCM has increased (1, 2). Traditional therapies, such as chemotherapy, have had limited effects on patients with advanced melanoma (3). Fortunately, immune checkpoint inhibitors (ICIs) has revolutionized melanoma treatment by activating the patient’s immune system (4). Many melanoma patients have experienced favorable treatment outcomes from ICIs targeting cytotoxic T lymphocyte antigen 4 (CTLA-4), programmed death-1 (PD-1), and PD-ligand 1 (PD-L1) (5–10). Adoptive cell transfer (ACT) therapy, another immunotherapy strategy, has also yielded encouraging results in recent years (11). Melanoma patients undergoing combinatorial immunotherapy have shown 5-year overall survival rates of up to 52% (12). However, due to the high heterogeneity, a significant portion of patients fail to respond to immunotherapy, and some experience unwanted immune-related adverse events (13, 14). Therefore, identifying factors that predict immunotherapy response may improve the effectiveness of these treatments and prolong patient survival.

The success of immunotherapy in melanoma is likely linked to the high tumor mutational burden (TMB). This high TMB generates a large pool of neoantigens, potentially triggering robust anti-tumor immune responses (15). However, despite the association between high TMB and inferred neoantigen load with overall survival, their connection to observed immunotherapy efficacy remains inconsistent (16). Other proposed biomarkers include mismatch-repair deficiency, CTLA-4 expression, and PD-1/PD-L1 status (17, 18). While these markers are already used clinically, they have limitations (19). The advent of bulk sequencing has facilitated “omics” studies to identify tumor characteristics predictive of immunotherapy response. For example, one study reported the expression level of PRRT3-AS1 as a biomarker for prognosis and immunotherapy response (20). Another study linked increased activity in the glycolysis/gluconeogenesis pathway to a positive response (21). Additionally, multi-gene signatures based on specific signaling pathways like inflammasome, m^6^A modification, and ferroptosis have been developed (22–24). However, their widespread clinical application is hampered by limitations in modeling methods and the typically small size of the datasets used.

Current clinical practice in melanoma relies heavily on the clinicopathological TNM staging system, which categorizes patients based on tumor size, lymph node involvement, and metastasis. However, this system falls short of capturing the full picture of a patient’s condition. Melanoma exhibits high inter- and intra-tumor heterogeneity, meaning patients at the same stage can have vastly different prognoses. An ideal biomarker would consistently reflect tumor biology across all patients, but the TNM system focuses solely on anatomical features. To address this limitation, researchers have explored multigene panels as predictive signatures that account for the biological diversity of melanoma (25–27). These approaches hold promise for more accurate risk assessment and treatment decisions. However, clinical application remains hindered by several factors, such as insufficient data, suboptimal machine learning methods, insufficient validation across diverse patient populations, and the absence of robust clinical testing.

**Figure 1:**
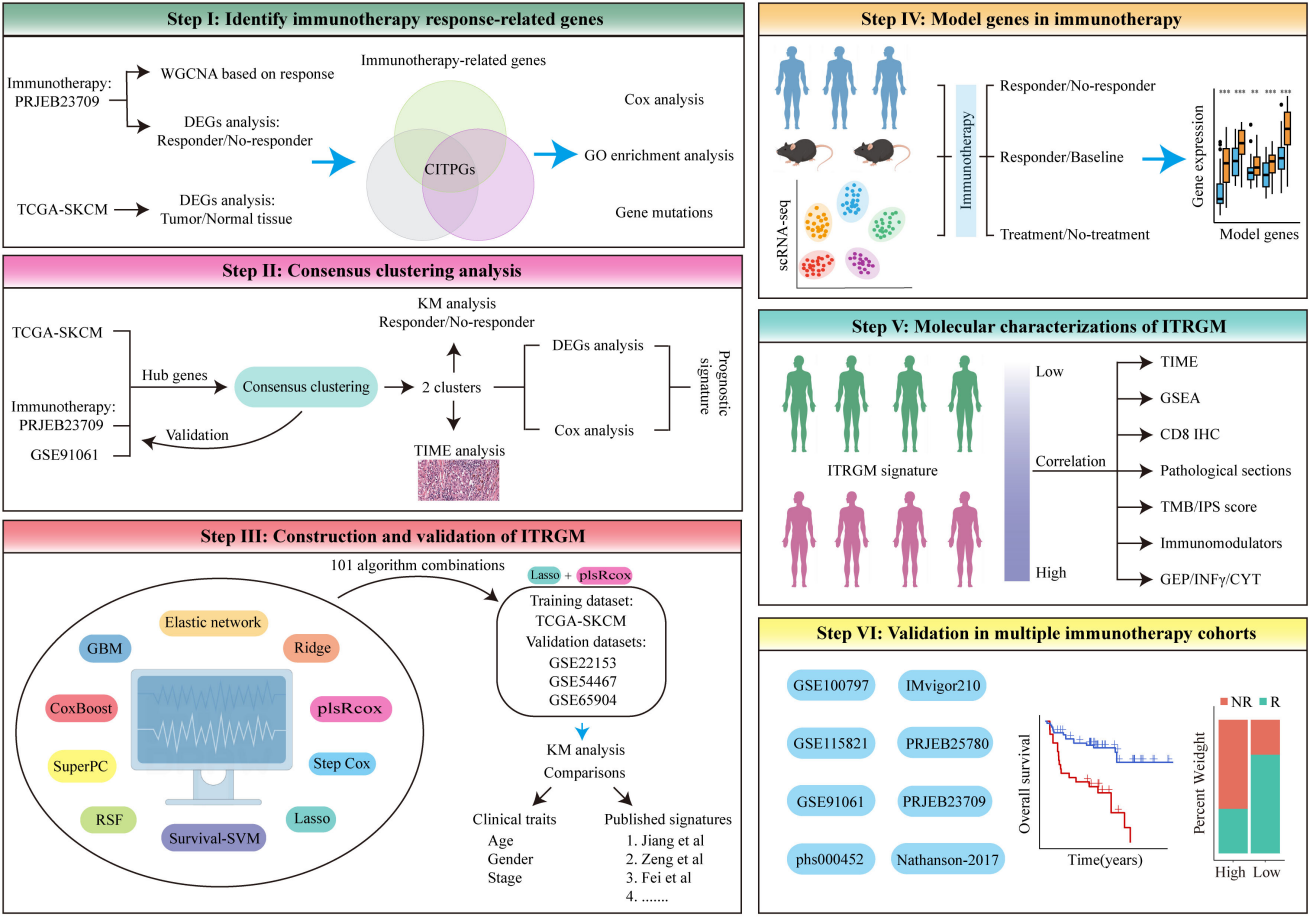
The flowchart of the analysis in this study comprises six different modules, Step I-VI. See the Methods section for details.

**Figure 2:**
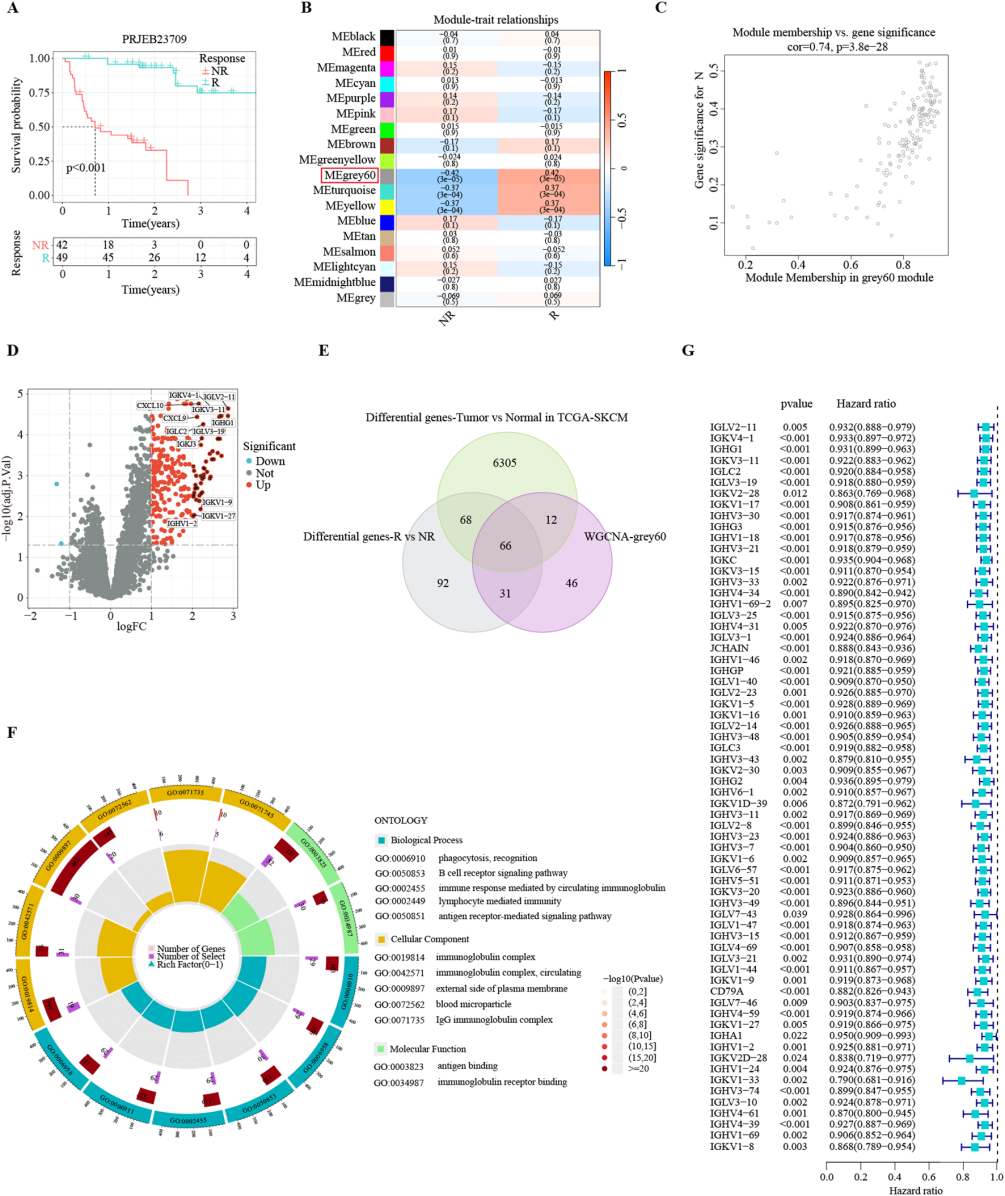
Identification of consensus immunotherapy prognostic genes (CITPGs). **(A)** Kaplan-Meier survival analysis based on immunotherapy responders and non-responders in PRJEB23709 dataset, indicating a favorable prognosis for immunotherapy responders. R (green line) represents responder. NR (red line) represents non-responder. **(B)** Correlation of 18 gene modules with response and non-response to immunotherapy, with the highest correlation for the Grey 60 module. **(C)** Gene correlation scatter plot showing the relationship between module membership (MM) and gene significance (GS) in the grey60 module. **(D)** Volcano plot showing DEGs between immunotherapy responders and non-responders in the PRJEB23709 dataset. Red circles with black dots indicate genes with more than 4 times upregulation. **(E)** Venn Diagram demonstrating the intersection of the genes in the grey60 module, DEGs between immunotherapy responders and non-responders, and DEGs between melanoma normal (GTEX) and tumor tissues (TCGA-SKCM). A total of 66 genes were obtained, termed CITPGs. **(F)** GO enrichment analysis based on the 66 CITPGs, demonstrating that the CITPGs is enriched to immune-related pathways. **(G)** Univariate Cox regression analysis of the 66 CITPGs based on overall survival (OS) in TCGA-SKCM dataset, showing that all 66 genes in the CITPGs were associated with better prognosis.

To develop a more reliable predictive biomarker for immunotherapy in melanoma, we constructed and rigorously validated the immunotherapy-related gene model (ITRGM) signature in this study. The construction process can be divided into three steps, corresponding to Steps I-III (**Figure 1**). In Step I, we first identified a consensus of 66 hub genes associated with immunotherapy, termed consensus immunotherapy prognostic genes (CITPGs). These 66 hub genes represent the intersection of 3 gene sets (**Figure 1** Step I, **Figure 2E**). The 3 gene sets are: (1) genes identified by WGCNA (weighted correlation network analysis) for gene pattern identification, (2) DEGs between immunotherapy responders and non-responders, and (3) DEGs between melanoma tumor tissues and normal tissues. The purpose of constructing these 66 hub genes, or CITPGs, is to identify genes are abnormally expressed in tumor tissues and also associated with immune responses. In Step II, we utilized CITPGs and performed consensus clustering analysis to refine the prognostic signature, which was then used in Step III. In Step III, we developed a seven-gene artificial intelligence-derived prognostic signature, the immunotherapy-related gene model (ITRGM), using 101 machine-learning algorithm combinations (28, 29).

During the validation phase, we demonstrated the strong correlation of ITRGM with the immune landscape across multi-omics levels (scRNA-seq, pathology sections, SNP, IHC, transcriptome). All seven genes in the ITRGM signature are all up-regulated in immunotherapy responders (**Figure 1** Step IV, section 3.6). Additionally, the ITRGM signature was shown to play a significant role in tumor immunity and the tumor microenvironment (**Figure 1** Step V, section 3.7). Importantly, we validated the predictive performance of the ITRGM signature in 4 immunotherapy-untreated melanoma cohorts and 8 immunotherapy clinical cohorts (**Figure 1** Step VI, section 3.8), demonstrating its ability to distinguish “immune-hot” tumors from “immune-cold” tumors and to predict prognosis and immunotherapy response in melanoma patients. Furthermore, beyond what is depicted in **Figure 1**, we also tested the correlation between GBP5 (one of the ITRGM signature genes) and CD8 (section 3.9) and evaluated the ITRGM signature’s relevance in chemotherapy efficacy (section 3.10). This work provides ITRGM as a stable and robust signature for predicting prognosis and response to immunotherapy in melanoma patients, potentially aiding in the clinical management of melanoma for improved clinical outcomes.

## 2 Materials and methods

### 2.1 Description of workflow

The workflow in our study is illustrated in **Figure 1**. In step I, we first developed a melanoma classification system designed to predict patient responses to immunotherapy from a multi-omics perspective. We utilized the melanoma immunotherapy cohort PRJEB23709, applying weighted gene co-expression network analysis (WGCNA) to identify gene modules associated with immune responses between immunotherapy responders and non-responders, alongside differential gene expression analysis to pinpoint genes that differed between these groups. The GEPIA database further provided information on differentially expressed genes (DEGs) in melanoma tumors versus normal tissues. From the intersection of these three gene sets (Genes in modules most related to immunotherapy response identified by WGCNA, DEGs between immunotherapy responders and non-responders, and DEGs between melanoma tumor tissues and normal tissues), we identified a list of 66 melanoma immunotherapy-related genes, termed consensus immunotherapy prognostic genes (CITPGs) (**Supplementary Table S1**). In step II, through consensus clustering, we determined that these CITPGs could effectively categorize melanomas into CITPG-high and CITPG-low groups and predict immunotherapy efficacy. To refine the melanoma classification system, in step III, we identified prognostically relevant DEGs between these groups (**Supplementary Table S2**) and developed a novel immunotherapy-related gene model (ITRGM) using a machine learning framework that integrated 10 algorithms and 101 combinations. In step III, IV and V, we assessed the ITRGM’s prognostic accuracy across multiple cohorts, compared its performance with existing models, and investigated the relationship between model genes and immunotherapy through transcriptome analysis, hematoxylin and eosin (H&E) pathology sections, and tumor tissue immunohistochemistry. Ultimately, in step VI, verification across various immunotherapy cohorts confirmed that the ITRGM reliably predicts tumor response to immunotherapy and patient prognosis.

### 2.2 Data acquisition and preprocessing

In total, data from 1808 cancer patients across 16 independent public cohorts were accessed in this study. RNA-seq data to extract FPKM values, clinical data, and mutation annotation format (MAF) files for TCGA-SKCM were downloaded from the GDC database (https://portal.gdc.cancer.gov/). We excluded samples with incomplete or missing clinical information, resulting in a cohort of 459 TCGA-SKCM patients. Three melanoma transcriptome data with clinical information were downloaded from Gene Expression Omnibus (GEO, http://www.ncbi.nlm.nih.gov/geo): GSE54467 (N = 79), GSE69504 (N = 214), and GSE22153 (N = 57).

To test the efficiency of the ITRGM signature in predicting the immunotherapy response of patients, we obtained 7 clinical cohorts that received immunotherapy from the TIGER database (http://tiger.canceromics.org/#/immuneResponse): GSE91061, PRJEB23709, GSE100797, Nathanson_2017, phs000452, STAD-PRJEB25780, and GSE115821, and 1 clinical cohort (IMvigor210) with “IMvigor210CoreBiologies” R package (30). The above transcriptome data were transformed from FPKM to log_2_(FPKM+1). The scRNA-seq dataset “SKCM_GSE115978_aPD1” was analyzed directly with the TISCH2 website (http://tisch.comp-genomics.org/home/). The transcriptional dataset GSE243238 (N = 17) of acral melanoma with CD8 immunohistochemistry data was downloaded from GEO. The expression data for genes in the ITRGM signature in two melanoma mouse immunotherapy cohorts (GSE109485 and GSE149825) were downloaded from the TISMO website (http://tismo.cistrome.org/). To assess the expression levels of model genes, the immunohistochemistry (IHC) images of model genes were downloaded from the Human Protein Atlas (HPA) database (https://www.proteinatlas.org/). To compare the expression levels of GBP5 in melanoma and normal skin tissues, the GSE15605 and GSE114445 datasets were downloaded from the GEO database. Details of all the datasets used in this study are listed in **Supplementary Table S3**.

### 2.3 Differential gene expression analysis and weighted gene co-expression network analysis

Differentially expressed genes (DEGs) between melanoma tumor tissue (TCGA-SKCM) and normal tissue (GTEx) were directly obtained from GEPIA2 (http://gepia2.cancer-pku.cn/#index). Genes with |log_2_FC| > 1 and FDR < 0.05, between responders and non-responders in the PRJEB23709 dataset, were selected using the limma package.

To gain insight into highly correlated gene clusters (modules) in the PRJEB23709 melanoma immunotherapy dataset, WGCNA analysis was performed using the R package “WGCNA” (31). First, the optimal soft threshold β was determined to construct a scale-free network. Next, we converted the weighted adjacency matrix into a topological overlap matrix (TOM) and calculated the dissimilarity (dissTOM). The dynamic tree-cut approach was used for clustering genes and module identification. Finally, we selected the gene module with the highest correlation with the immunotherapy response for subsequent study. To assess the correlation between gene significance (GS) and module membership (MM) in the gene module, the Pearson correlation coefficient and corresponding *P* values were calculated using the WGCNA package.

### 2.4 Consensus clustering

To identify the genes commonly detected as immunotherapy predictive genes in melanoma, we extracted three gene sets to compare their intersections: the modular genes most relevant to the immunotherapy response identified by WGCNA analysis, DEGs between immunotherapy responders and non-responders in the PRJEB23709 cohort, and DEGs between melanoma tumor samples and normal tissues from TCGA-SKCM. This resulted in the identification of 66 hub genes as CITPGs. Subsequently, consensus clustering of TCGA-SKCM patients was performed based on these CITPGs using the R package “ConsensusClusterPlus” (32). The optimal number of clusters (k) was determined to be 2, based on the cumulative distribution function. Furthermore, PCA analysis and Kaplan-Meier analysis were performed between the two clusters using the R packages “ggplot2” and “survival”, respectively. Univariate Cox regression analysis of the 66 CITPGs was performed using the R package “survival”.

### 2.5 Construction and validation of ITRGM model

The overall flowchart of this method is shown in Step III of **Figure 1**. Firstly, we obtained DEGs between the two clusters that were significantly associated with prognosis in all four melanoma datasets (TCGA-SKCM, GSE54467, GSE69504, and GSE22153). Subsequently, models were fitted based on these genes. The TCGA-SKCM dataset was used as the training set, while GSE22153, GSE54467, and GSE59455 were used as the validation sets. We incorporated ten machine learning algorithms: elastic net (Enet), ridge regression, partial least squares regression for Cox (plsRcox), stepwise Cox, lasso, survival support vector machine (survival-SVM), random survival forest (RSF), supervised principal components (SuperPC), CoxBoost, and generalized boosted regression modeling (GBM). A tenfold cross-validation network was employed to evaluate 101 combinations of these algorithms in the TCGA-SKCM training set for variable selection and model construction (28, 29). For each model, we evaluated its C-index across all the training and validation sets. Finally, we ranked the average C-index of all models and selected the model (Lasso+plsRcox) with the highest average C-index to develop ITRGM for predicting melanoma response to immunotherapy and prognosis.

In the Lasso+plsRcox machine learning algorithm, Lasso regression was initially performed using the survival duration and survival status of each patient along with the expression matrix of the 44 genes. During this process, each gene was assigned a regression coefficient. Genes with coefficients close to zero, indicating a weak relationship with survival, were removed from the model. Ultimately, seven genes—GBP5, HLA-DPB1, XBP1, CD40, GBP1, CXCL10, and TNFSF13B—were identified with non-zero coefficients. These genes were then used in the plsRcox algorithm to compute risk scores. The code for the Lasso+plsRcox model is available on GitHub: https://github.com/YuBestLab/YuBestLab.github.io/tree/101-machine-learning-algorithm.

In constructing the meta-cohort (TCGA-SKCM, GSE22153, GSE54467, GSE69504), we used the “limma” and “sva” R packages to merge the four cohorts after removing batch effect. In addition, all cohorts were normalized using the “standarize.fun” function for ITRGM score calculation. This standardization is important for data analysis and machine learning algorithms, as many algorithms do not perform well with data on different scales. By standardizing the data, we ensure it is comparable and has better numerical stability, thus improving the performance and effectiveness of the algorithms. Univariate Cox regression analysis, multivariate Cox regression analysis, and calibration curve analysis of ITRGM were performed with the “survival” R package.

### 2.6 Functional enrichment analysis

The R packages “clusterprofile” (33) and “maftools” (34) were used to perform Gene Ontology (GO) enrichment analysis for the 66 CITPGs and to analyze MAF files, respectively. Additionally, the R package “clusterprofile” was used to perform Gene Set Enrichment Analysis (GSEA) based on the gene set “c2.cp.kegg.v2023.1.Hs.symbols.gmt”. GSEA on GBP5 was performed using LinkedOmics (https://linkedomics.org/#/) (35).

### 2.7 Assessment of tumor immune microenvironment characteristics

The TCGA-SKCM immune microenvironment was evaluated for StromalScore, ImmuneScore, and ESTIMATEScore using R with the ESTIMATE algorithm (36). The ssGSEA algorithm was utilized to evaluate 28 immune cell infiltrations and 29 immunofunctional activities in TCGA-SKCM for heat mapping. The response of TCGA-SKCM to immunotherapy was predicted with the TIDE online tool (http://tide.dfci.harvard.edu/) (37, 38). The TIP website (http://biocc.hrbmu.edu.cn/TIP/index.jsp) (39) was used to obtain scores for the cancer-immunity cycle, which consists of seven steps, in TCGA-SKCM patients. The gene lists for Immunoinhibitor, MHC, and Immunostimulator were downloaded from TISIDB (http://147.8.185.80/TISIDB/) (40). The IPS scores were downloaded from TCIA (https://tcia.at/home). Additionally, immune cell infiltration data were obtained from the TIMER2 website (http://timer.cistrome.org/) (41–47) using multiple algorithms including TIMER, CIBERSORT, quanTIseq, xCell, MCP-counter, and EPIC. After obtaining the infiltration scores, we performed a Spearman correlation analysis between the risk scores and the infiltration scores to obtain the correlation coefficients. These data were also used for Kaplan-Meier analysis. Correlation between GBP5 expression levels and CD8^+^ T cell infiltration in 32 cancer types were performed using the TIMER2 website.

### 2.8 Histological examination of the TCGA-SKCM samples

Histologic data (annotation.tsv) for TCGA-SKCM (N = 63) were downloaded from Bagaev et al. (48). Semiquantitative scores of lymphocytes for melanoma were obtained using a 5-grade system (0-4) from the dataset (48). Tumor cellularity was defined as the percentage of tumor cells detected in the sample slides out of all cells present. Pathology sections stained with hematoxylin and eosin (H&E) were provided by the GDC database (https://portal.gdc.cancer.gov/).

### 2.9 Multiplex immunofluorescence

Based on bioinformatics studies, tissue microarrays containing 17 cases of melanoma and 18 cases of normal skin tissue were obtained from Shanghai Wellbio Technology Co., Ltd. To confirm the expression of GBP5 in melanoma and its association with CD8^+^ T-cell infiltration, the slices underwent sequential treatment with eco-friendly dewaxing solution I (three times for 10 min each), anhydrous ethanol (four times for 5 min each), and were washed with distilled water. High-pressure antigen retrieval was performed, followed by natural cooling. The slides were placed in PBS (pH 7.4) on a decolorization shaker and washed three times for 5 min each. After drying the sections, a circle was drawn around the tissue with a histochemical pen, and 3% BSA was added to block the sections for 30 min.

The mixture of the first primary antibody (GBP5, Abcam: AB313390) and the second primary antibody (CD8, Servicebio: GB12068) was then added. The prepared primary antibodies were applied dropwise, and the sections were incubated flat in a wet box at 4°C overnight. After incubation, the slides were washed in PBS (pH 7.4) three times for 5 min each on a decolorization shaker. The corresponding secondary antibodies (Alexa Fluor 488-conjugated goat anti-rabbit IgG for GBP5 and Cy3-conjugated goat anti-mouse IgG for CD8) were added, and the slides were incubated for 50 min at room temperature, protected from light. After incubation, the slides were washed three times in PBS (pH 7.4) for 5 min each on a decolorization shaker, followed by the addition of autofluorescence quencher B solution for 5 min, and rinsed under running water for 10 min. Images were captured using specific excitation and emission wavelengths for DAPI (excitation 380 nm, emission 420 nm), Alexa Fluor 488 (excitation 480 nm, emission 535 nm), and Cy3 (excitation 535 nm, emission 590 nm).

Fluorescence intensity was quantified using ImageJ or Fiji by calculating the mean intensity of the selected region of interest (ROI) and subtracting the mean intensity of the background ROI. The mean intensity of 20-45 ROIs, each approximately 100 μm × 100 μm in size, was measured and then averaged to determine the fluorescence intensity of each sample.

### 2.10 Analysis of drug sensitivity

The R package “oncopredict” (47) was utilized to predict the chemosensitivity of TCGA-SKCM patients using the expression file data and drug response information in Genomics of Drug Sensitivity in Cancer (GDSC2) as a reference. Samples were divided into high and low risk groups based on the median risk score determined by the ITRGM signature. Differential sensitivity to drugs between these groups was examined using the Wilcox test. Correlation coefficients between drug sensitivity scores and risk scores were analyzed using the Spearman method.

### 2.11 Statistical analyses

All statistical analyses were performed using R software (R version 4.2.3) or GraphPad Prism 8.0.2. Kaplan-Meier analyses and log-rank tests between two groups were conducted with the R package “survival”. Prognostic genes were identified through univariate Cox analysis. Correlation analyses were performed using either Spearman or Pearson methods as appropriate. Differences between two groups were assessed using the Wilcoxon test or unpaired *t*-tests. *P* < 0.05 was considered statistically significant. * *P* < 0.05; ** *P* < 0.01; *** *P* < 0.001.

## 3 Results

### 3.1 Identification of key genes related to immunotherapy response in melanoma

The application of immunotherapy has emerged as a breakthrough in melanoma treatments. The PRJEB23709 cohort, an immunotherapy dataset for melanoma, offers high-quality transcriptomic data, along with survival times and responses to treatment. In this immunotherapy cohort of metastatic melanoma, the survival of immunotherapy responders was substantially prolonged (**Figure 2A**). To identify gene modules strongly correlated with immunotherapy response in melanoma, we conducted WGCNA analysis with the PRJEB23709 cohort to identify patterns of genes. We utilized the soft threshold parameter β = 5 to ensure the construction of a scale-free gene network (**Supplementary Figure S1A**). Subsequently, 18 gene modules were established, as shown by the dendrogram of genes clustered via the dissimilarity measure. (**Supplementary Figure S1B**). The Grey 60 module showed the strongest association with immunotherapy response efficiency (**Figure 2B**). The relationship between gene significance (GS) and module membership (MM) in the Grey 60 module showed a significant positive correlation (cor = 0.74, *P* = 3.8e-28), suggesting the positive association of this gene module with immunotherapy response (**Figure 2C**).

To identify the list of genes most reliably associated with melanoma as consensus immunotherapy prognostic genes (CITPGs), we compared three sets of gene lists. First, employing stringent criteria (|log_2_FoldChange| > 1 and FDR < 0.05), we found 257 DEGs between immunotherapy responders and non-responders in the PRJEB23709 cohort (**Figure 2D**). Second, we acquired 6457 DEGs between melanoma tumors (TCGA-SKCM) and normal tissues from GEPIA2. Finally, we compared the genes in the Grey 60 module with these two gene sets (DEGs between immunotherapy responders and non-responders, DEGs between melanoma tumor tissues and normal tissues) and identified 66 genes as the CITPGs (**Figure 2E**; **Supplementary Table S1**). GO functional enrichment analysis showed that CITPGs were highly enriched in several immune-related pathways, such as phagocytosis, B cell receptor signaling, and immunoglobulin (**Figure 2F**). Next, we tested whether CITPGs are associated with the prognosis of melanoma patients through univariate Cox regression analysis using the TCGA-SKCM cohort. Remarkably, we found that all 66 genes in CITPGs showed a slight but significant reduction in the risk of the prognosis of melanoma patients (**Figure 2G**). We also analyzed the mutation status of the CITPGs in TCGA-SKCM cohort. The results showed that many mutations were present in CITPGs predominantly with missense mutations, suggesting a potential implication of CITPGs in melanoma progress (**Supplementary Figure S1C**).

### 3.2 Consensus clustering analysis of CITPGs

**Figure 3:**
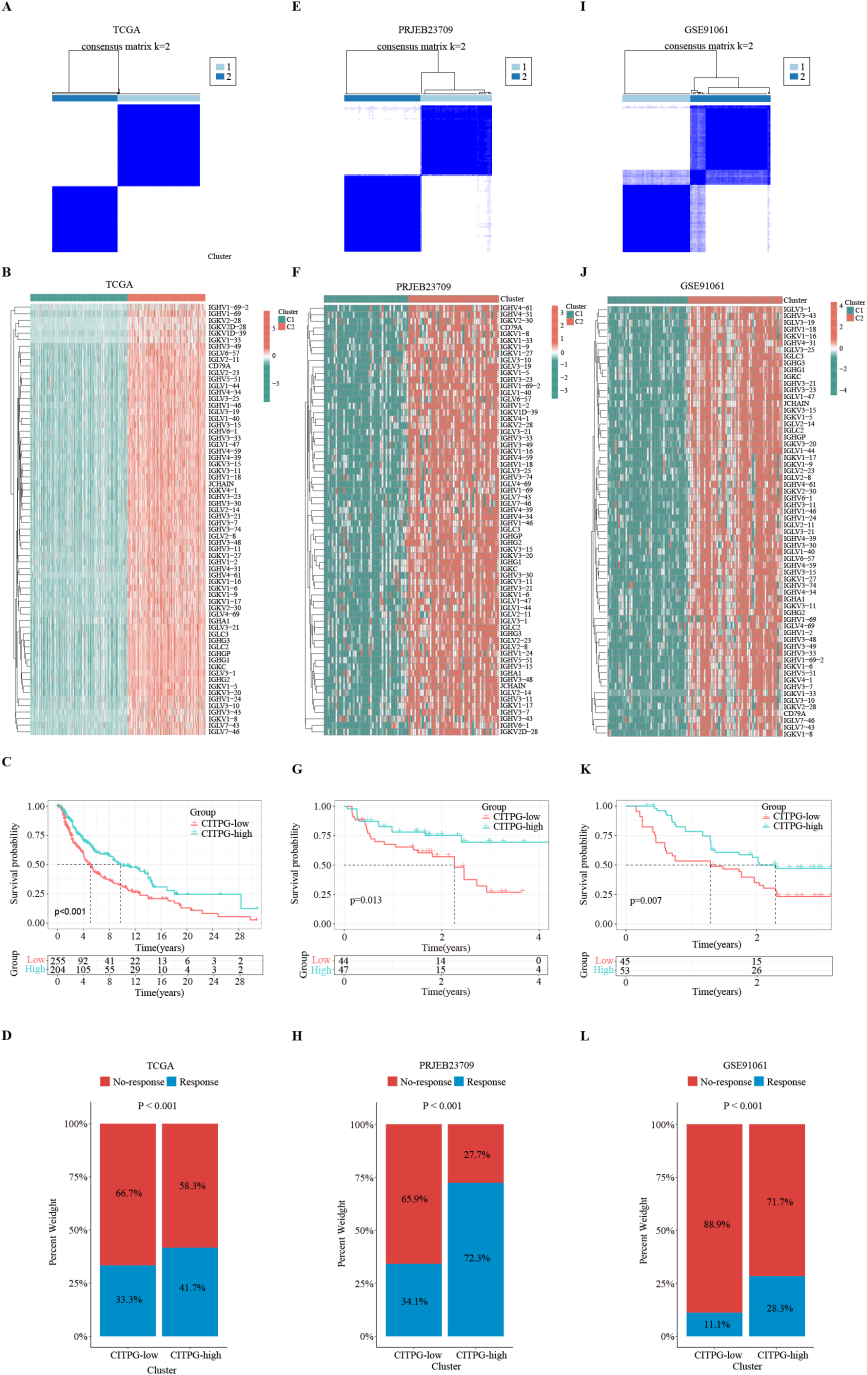
Identification of two distinct clusters with consensus clustering analysis. **(A, E, I)** Consensus clustering divides the patients in TCGA-SKCM, PRJEB23709, and GSE91061 datasets into two clusters, C1 and C2, based on the expression of 66 CITPGs. **(B, F, J)** The expression levels of 66 CITPGs in the two clusters in the TCGA-SKCM, PRJEB23709, and GSE91061 datasets, with Cluster2 (C2) showing higher expression. **(C, G, K)** Kaplan-Meier survival analysis on overall survival (OS) between two clusters, CITPG-low and CITPG-high, in the TCGA-SKCM, PRJEB23709, and GSE91061 datasets, showing a better prognosis for the CITPG-high group. **(D, H, L)** Stacked graphs showing the percentage of immunotherapy responders/non-responders between the CITPG-low and CITPG-high clusters in TCGA-SKCM, PRJEB23709, and GSE91061 datasets, The CITPG-high cluster exhibited higher response rates to immunotherapy in three different databases.

Consensus clustering, an unsupervised clustering method, is a common research method for classifying cancer subtypes. It allows for the distinction of samples into subtypes based on different histological datasets, thereby facilitating the analysis of the specificity of the different subtypes as well as the response to certain therapies. To identify distinct subgroups of TCGA-SKCM patients based on CITPG expression patterns, we employed consensus clustering. The optimal clustering stability was reached when the number of clusters (K) equals 2 (**Figure 3A**; **Supplementary Figures S1D, E**). Consequently, we divided the cohort into two subgroups (**Figure 3A**): one group with higher CITPG expression, referred to as the CITPG-high group (C2), and the other with lower CITPG expression, termed the CITPG-low group (C1) (**Figure 3B**). Principal component analysis (PCA) further confirmed the effectiveness of this classification (**Supplementary Figure S1F**). Notably, the CITPG-high group exhibited significantly better prognosis (**Figure 3C**).

Using the TIDE algorithm, we predicted the response of the TCGA-SKCM cohort to immunotherapy. The results revealed that the CITPG-high group had a significantly higher proportion of immunotherapy responders (**Figure 3D**). Subsequently, we assessed whether the expression pattern of CITPGs could serve as a valid predictive factor for melanoma immunotherapy. Consensus clustering analysis based on CITPG expression was applied to two melanoma cohorts (PRJEB23709, GSE91061) that underwent immunotherapy. Remarkably, both cohorts showed clear separation of patients into two groups. (**Figures 3E, F, I, J**; **Supplementary Figures S1D–F**). Importantly, the CITPG-high group exhibited better prognosis as well as a higher percentage of immunotherapy responses (**Figures 3G, H, K, L**), highlighting CITPGs as a promising feature for predicting prognosis in melanoma.

### 3.3 Analysis of the tumor immune microenvironment between the CITPG-high and CITPG-low groups

**Figure 4:**
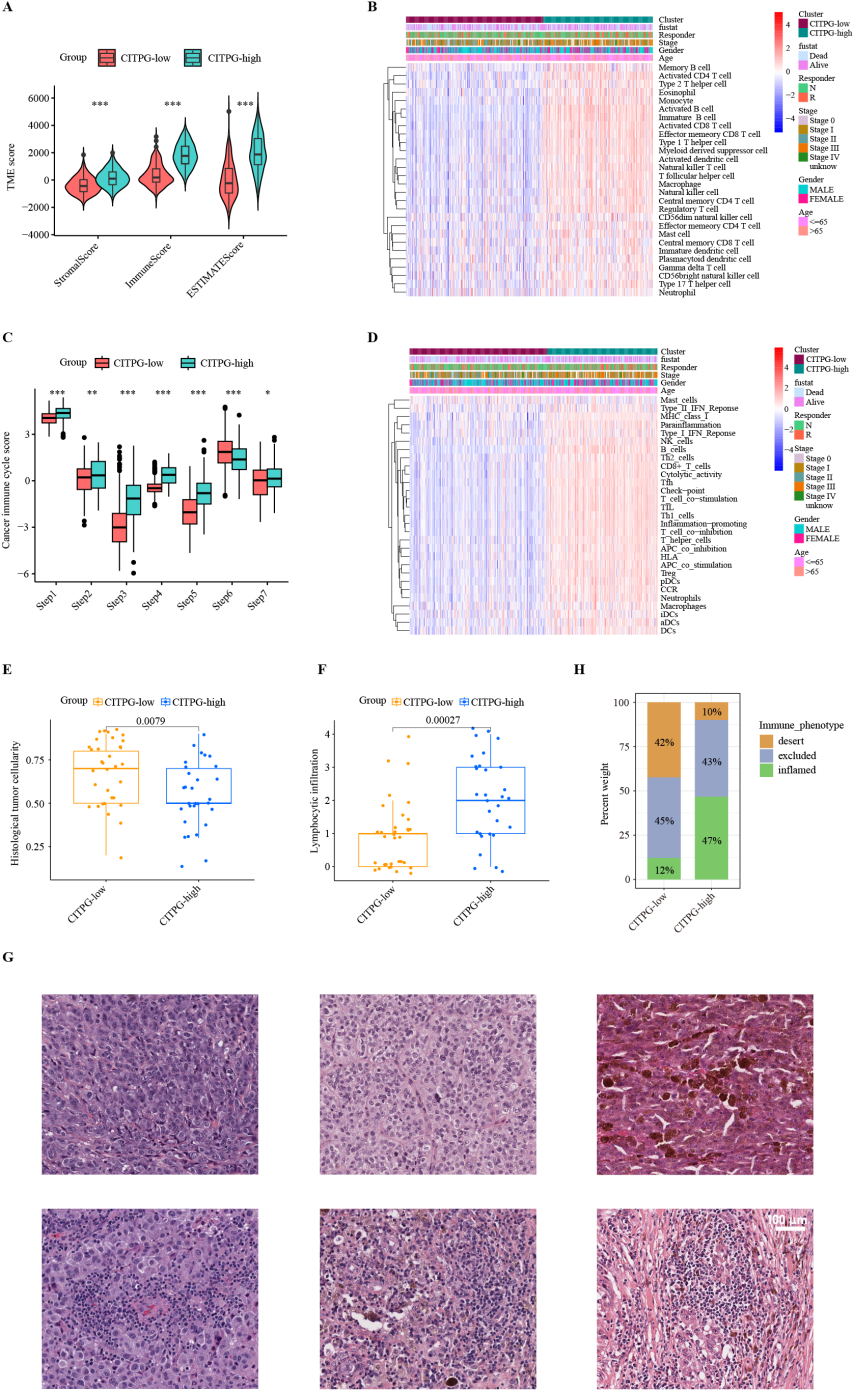
Characteristics of the tumor immune microenvironment (TIME) between two clusters, CITPG-low and CITPG-high. **(A, C)** Comparison of TME scores **(A)** and cancer immune cycle scores **(C)** between the CITPG-low and CITPG-high clusters in TCGA-SKCM dataset, showing that the CITPG-high cluster is associated with higher TME scores and overall higher cancer immune cycle scores *P < 0.05; **P < 0.01; ***P < 0.001 **(B, D)** The ssGSEA analysis indicates differences in 28 immune cell infiltrations and 29 immunofunctional activities between the CITPG-low and CITPG-high clusters in TCGA-SKCM. The CITPG-high cluster is associated with increased immune cell infiltration and enhanced immunofunctional activities. **(E, F)** Boxplots show histological tumor cellularity and lymphocytic infiltration based on histological examination of the TCGA-SKCM samples between the CITPG-low and CITPG-high clusters. The CITPG-high cluster is associated with lower tumor cellularity and higher lymphocytic infiltration **(G)** Representative hematoxylin-eosin (H&E) histological images of the CITPG-low and CITPG-high clusters in TCGA-SKCM reveal that the CITPG-high cluster is associated with higher lymphocytic infiltration. Scale bars in all panels are 100 μm. **(H)** Stacked graphs show the percentage of desert, excluded, and inflamed immune phenotypes between the CITPG-low and CITPG-high clusters in TCGA-SKCM, with the CITPG-high cluster showing a higher percentage of inflamed immune phenotypes.

To explore the mechanisms underlying the disparate clinical outcomes observed in the two clusters based on CITPG expression, we analyzed the status of the tumor immune microenvironment (TIME) in the two CITPG groups of TCGA-SKCM. First, we utilized the ESTIMATE package (44) to infer the levels of stromal and immune cells in melanoma tumors. The results showed significantly higher TIME values of stromal, immune, and ESTIMATE scores in the CITPG-high group compared to the CITPG-low group (**Figure 4A**).

Next, we employed the single-sample GSEA (ssGSEA) algorithm to evaluate the infiltration levels of various types of immune cells in TCGA-SKCM. We found that almost all immune-related cells were significantly more infiltrated into the tumor in the CITPG-high group (**Figure 4B**). To gain insight into whether the increased infiltrated immune cells were functionally active for anti-cancer immune response, we analyzed the status of cancer immune cycle scores between the two groups using TIP (39). Consistently, we found that the CITPG-high group exhibited increased activity in steps related to antigen release (step 1), antigen presentation (step2), activation (step 3), T cell transfer (step 4), immune cell infiltration (step 5), and cancer cell killing (step 7), while decreased activity in T cell recognition (step 6) (**Figure 4C**). Additionally, the ssGSEA analyses revealed that overall, the CITPG-high group had significantly higher immunomodulatory activities compared to the CITPG-low group (**Figure 4D**). These results indicate that the CITPG-high group likely represents “immune-hot” tumors, while the CITPG-low group may correspond to “immune-cold” tumors.

To further confirm the relationship between CITPG expression pattern and anti-cancer-immune response, the histological phenotypes associated with CITPG expression patterns were examined using hematoxylin and eosin (H&E)-stained sections of melanoma from TCGA-SKCM (N = 63). In line with our prediction, the CITPG-high melanomas had lower malignant cell cellularity and higher lymphocytic infiltration, indicating higher levels of infiltrated immune cells (**Figures 4E–G**). Additionally, the quantitative histopathological analysis of TCGA-SKCM revealed that CITPG-high melanomas showed enrichment of the immune-inflamed histological phenotype, but the CITPG-low group was characterized by an increased immune-desert phenotype (**Figure 4H**).

### 3.4 Integrative construction of immunotherapy-related gene model

**Figure 5:**
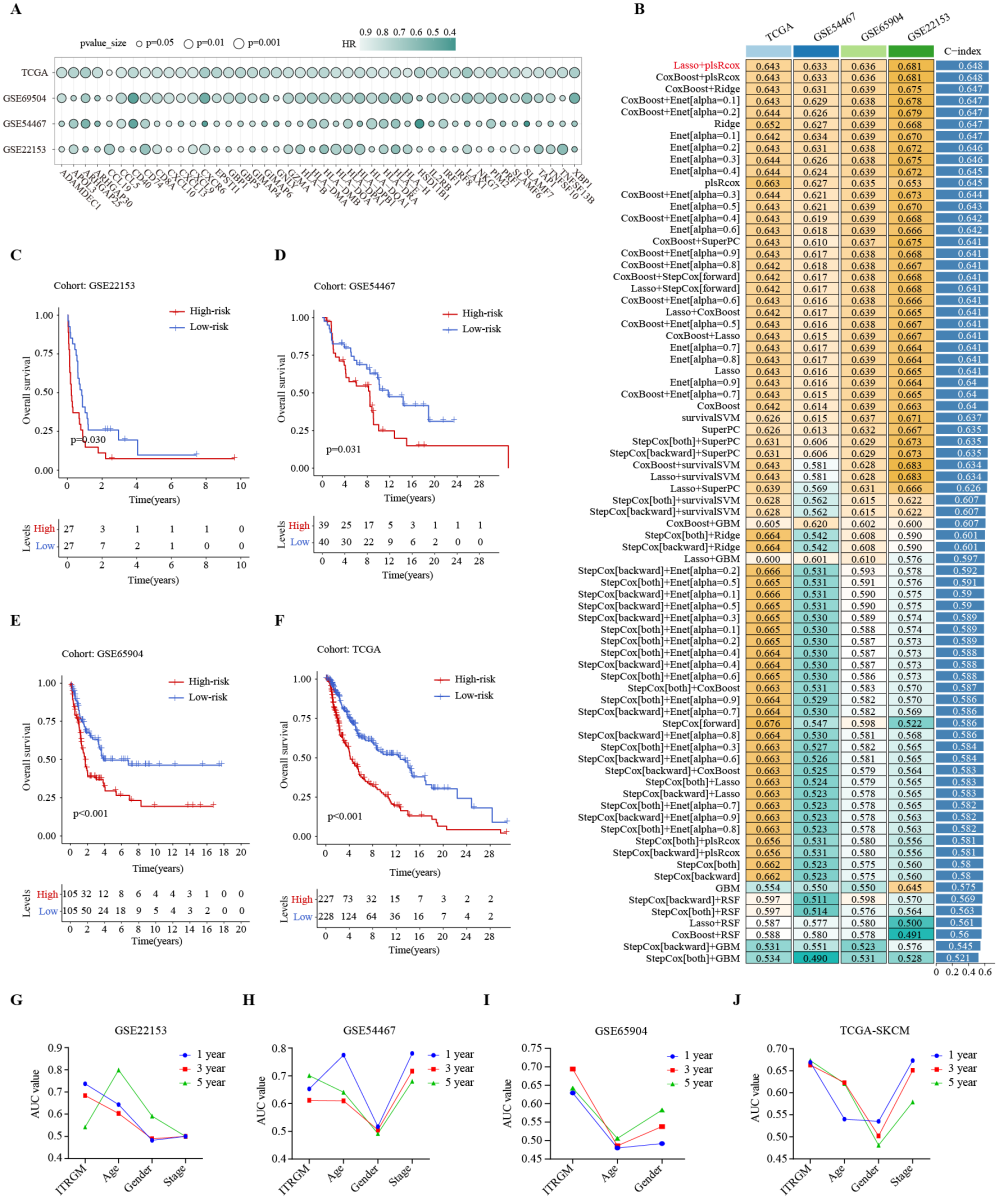
Development of immunotherapy-related gene model (ITRGM) signature via 101 machine learning combinatorial algorithms. **(A)** Univariate Cox regression analysis demonstrating DEGs significantly associated with prognosis in all the TCGA-SKCM, GSE69504, GSE54467, and GSE22153 datasets. A total of 44 genes were identified as being associated with improved prognosis. The size of the circle represents the p-value and the shade of the color represents the HR-value. **(B)** A total of 101 kinds of prediction models via LOOCV framework and further calculated the C-index of each model across all validation datasets. The Lasso+plsRcox model demonstrated the highest C-index. Numbers represent the C-index of the corresponding model in the corresponding cohort (GSE22153, GSE54467, GSE65904, TCGA-SKCM). Numbers in the rightmost bars represent the average C-index of the corresponding model in the four cohorts. **(C–F)** Kaplan–Meier curves based on OS according to the ITRGM signature in GSE22153, GSE54467, GSE65904, and TGCA-SKCM datasets revealed that the low-risk group was associated with better prognosis. **(G–J)** The area-under-the-curve (AUC) values of the receiver operating characteristic (ROC) curves for 1, 3, and 5 years, comparing ITRGM signature and certain clinical traits in GSE22153, GSE54467, GSE65904, and TGCA-SKCM datasets, demonstrated that the ITRGM signature consistently achieved better and more reliable AUC values.

The CITPGs comprise 66 genes, which is still too many to be practical for analysis. To develop a more streamlined and robust immunotherapy-related gene model (ITRGM), we screened for DEGs between CITPG-high and CITPG-low groups within the TCGA-SKCM cohort and identified 566 DEGs. We then incorporated three additional melanoma cohorts (GSE22153, GSE54467, GSE69504) and performed univariate Cox regression analysis on the 566 DEGs across these four datasets. This analysis identified 44 consensus genes significantly associated with patient prognosis (**Figure 5A**; **Supplementary Table S2**). The benefit of integrative procedures is the ability to fit a mode with consistent performance for SKCM prognostics based on multiple machine learning algorithms and their combinations. Furthermore, the combination of algorithms can reduce the dimensionality of the variables, making the models more simplified and translatable. The TCGA-SKCM cohort comprises a wealth of clinical data on melanoma patients, along with genomic variants, mRNA expression, H&E pathology sections, and other valuable resources, making it an invaluable source of data for cancer researchers. Therefore, using gene expression profiles of these 44 genes, we integrated 101 machine learning algorithm combinations (28, 29) on the TCGA-SKCM cohort as the training set to construct the optimal algorithm for ITRGM and calculated the average C-index of each combination. The algorithmic combination of “Lasso+plsRcox” exhibited the highest average C-index, thus being selected as the final model. This approach identified seven genes (CD40, GBP5, HLA-DPB1, XBP1, CXCL10, GBP1, TNFSF13B) as model genes for ITRGM (**Figure 5B**).

To explore the relationship between ITRGM and patient survival outcomes, we divided melanoma patients into high and low-risk groups based on the risk score acquired by ITRGM via the “Lasso+plsRcox” method. The risk score is negatively correlated with expression levels of model genes, meaning that higher expression of model genes indicates a lower risk score. Kaplan-Meier survival analysis indicated that low-risk melanoma patients had better prognoses in the GSE22153, GSE54467, GSE69504, and TCGA cohorts (**Figures 5C–F**). Compared to commonly used clinical prognostic traits such as age, gender, and cancer stage, the ITRGM showed superior and more reliable prognostic predictive power, as indicated by the area-under-the-curve (AUC) values of the receiver operating characteristic (ROC) curves (**Figures 5G–J**).

### 3.5 Comparison of ITRGM with previously published signatures in melanoma

Recent advancements in high-throughput sequencing, big-data technologies, and machine-learning algorithms have facilitated the development of numerous prognostic and predictive signatures for immunotherapy in melanoma (48, 49). To better evaluate ITRGM as a prognostic biomarker, we retrieved published signatures related to immunotherapy in melanoma for a comprehensive comparison of predictive accuracy. Signatures using miRNAs were excluded as none of the cohorts we used included miRNA expression information.

**Figure 6:**
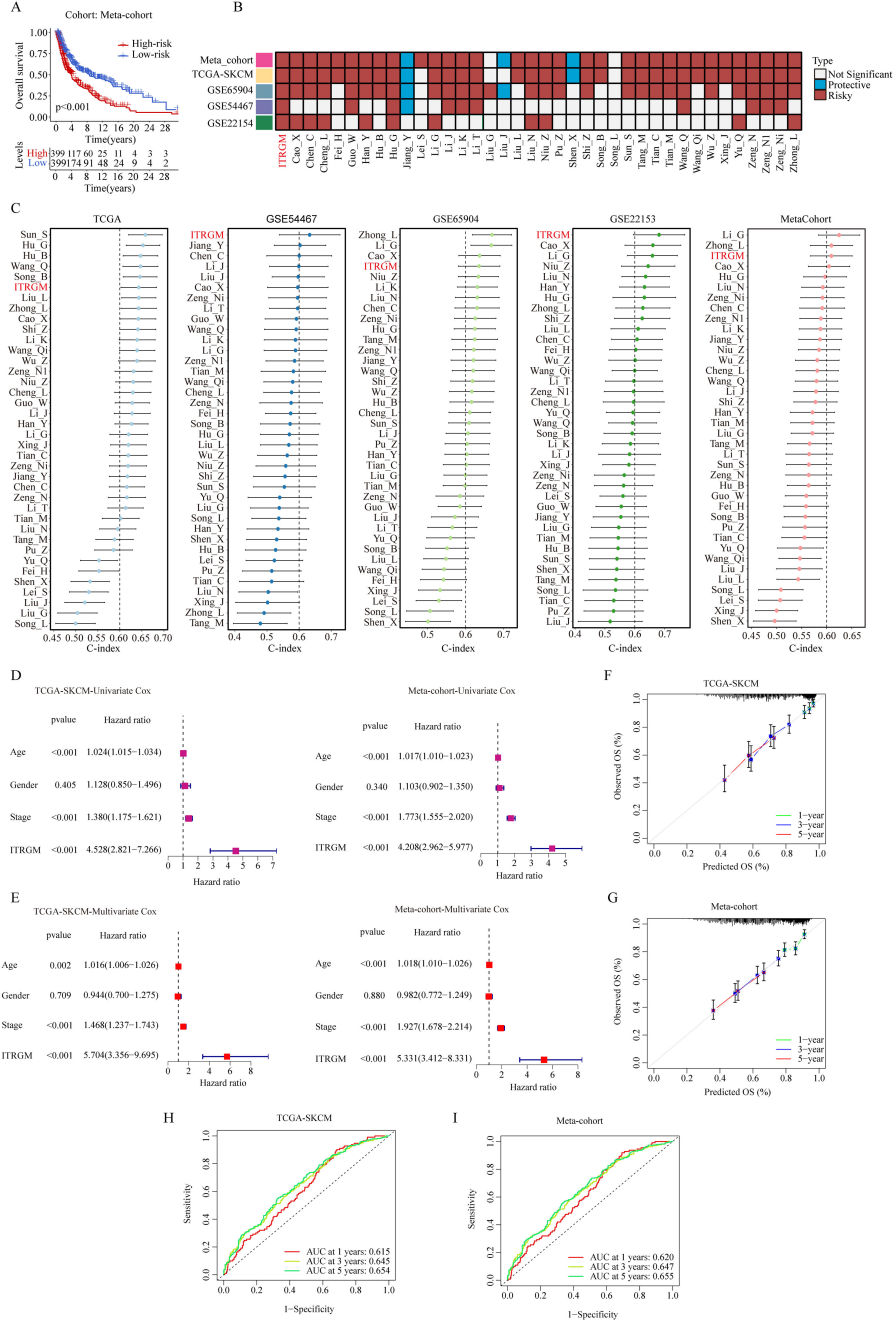
Comparison of ITRGM signature with 37 published signatures in melanoma. **(A)** Kaplan–Meier curves based on OS according to the ITRGM signature in the meta-cohort, revealed that the low-risk group was consistently associated with a better prognosis. **(B)** Univariate Cox regression analysis comparing ITRGM with 37 published signatures in melanoma. The light blue color denotes that the model is a significant protective factor for prognosis in the cohort, whereas the dark red color indicates that the model is a significant risk factor for prognosis. A white color signifies that the model does not have a significant association with prognosis in the cohort. **(C)** Comparison of C-indexes between the ITRGM signature and 37 published signatures in TCGA-SKCM, GSE54467, GSE65904, GSE22153, and the meta-cohort datasets demonstrated that the ITRGM signature consistently achieved a higher and more stable C-index overall. **(D, E)** Univariate **(D)** or multivariate **(E)** Cox regression analysis of ITRGM and several clinical traits (age, gender, stage) in TCGA-SKCM cohort (left) and the meta-cohort (right) demonstrated that ITRGM signature can serve as an independent prognostic factor. **(F, G)** Calibration curve for predicting 1-, 3-, and 5-year OS in the TCGA-SKCM cohort **(F)** and meta-cohort **(G)**. **(H, I)** The receiver-operator characteristic (ROC) analysis for predicting 1-, 3-, and 5-year OS in the TCGA-SKCM cohort **(H)**, and meta-cohort demonstrated the satisfactory predictive performance of the ITRGM signature **(I)**.

After removing the batch effect, we built a meta-cohort by combining four cohorts (TCGA-SKCM, GSE22153, GSE54467, GSE69504), which showed a consistent trend of the low-risk group having better prognosis (**Figure 6A**). Next, we conducted a comprehensive benchmark of our ITRGM alongside 37 published prognostic signatures. First, univariate Cox analyses of all models revealed that only our ITRGM and Hu_G models demonstrated consistent statistical significance of association with prognosis across the four independent cohorts and the meta-cohort (**Figure 6B**), demonstrating the stability of ITRGM.

Furthermore, we compared the C-index of ITRGM with other signatures. Remarkably, the ITRGM exhibited superior performance, achieving the highest accuracy in two cohorts, GSE22153 and GSE54467, and exhibiting comparable results with other top signatures in GSE65904, TCGA, and the meta-cohort (**Figure 6C**). Finally, the independent prognostic significance of ITRGM was evaluated through univariate and multivariate Cox analyses. The results demonstrated that ITRGM is a robust and independent prognostic factor (**Figures 6D, E**). The calibration curves and receiver-operator characteristic (ROC) curves showed that ITRGM exhibited satisfactory prediction performance in both the TCGA-SKCM training cohort and the meta-cohort (**Figures 6F–I**).

### 3.6 Roles of model genes in immunotherapy cohorts

**Figure 7:**
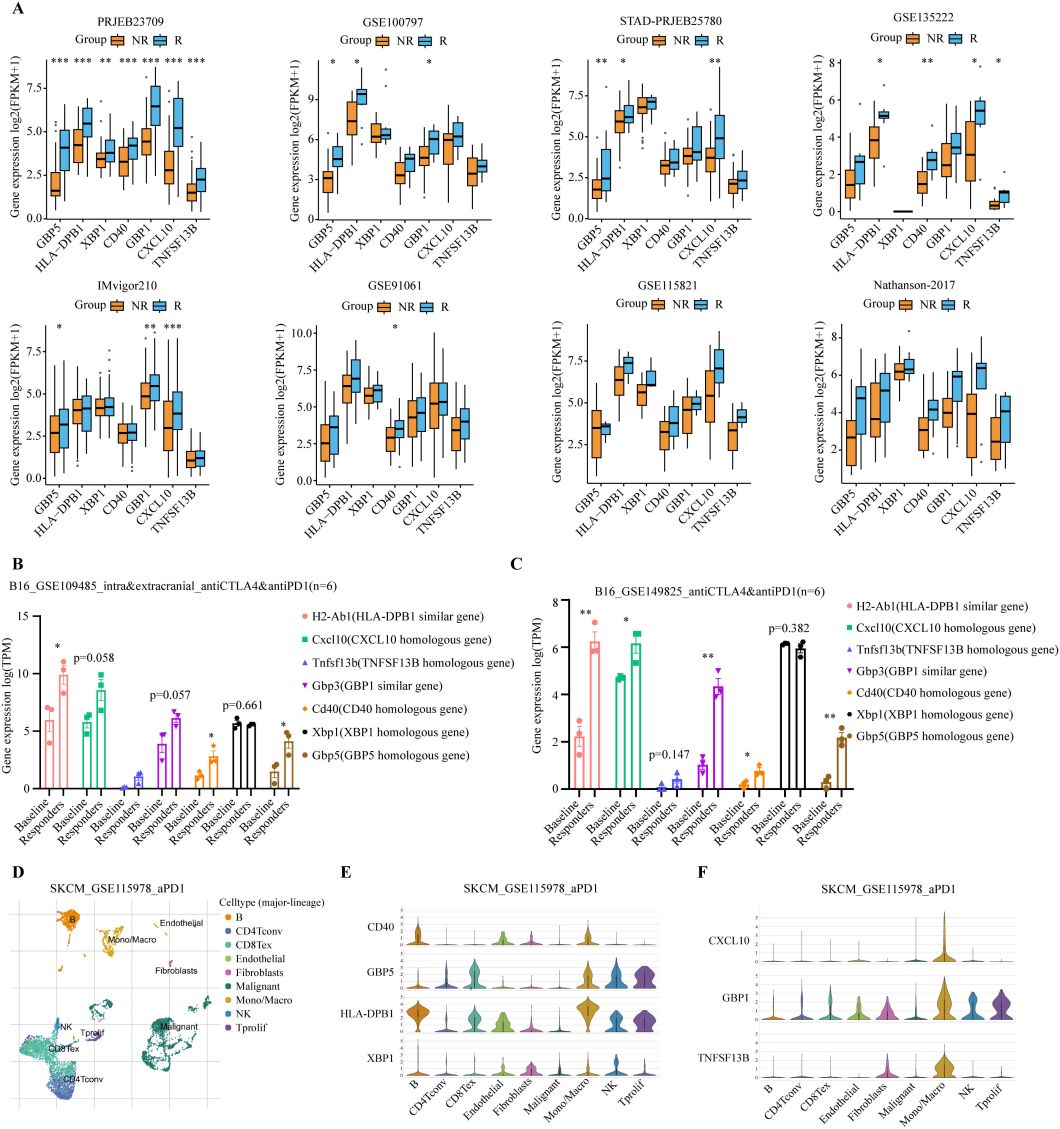
Analysis of expression levels of seven model genes in immunotherapy cohorts. **(A)** Expression levels of the 7 model genes were compared between immunotherapy responders (R, blue) and non-responders (NR, orange) in PRJEB23709, GSE100797, STAD-PRJEB25780, GSE135222, IMvigor210, GSE91061, GSE115821, and Nathanson-2017 immunotherapy datasets. Responders exhibited higher expression levels of the modeled genes. *P < 0.05; **P < 0.01; ***P < 0.001 **(B, C)** Expression levels of the 7 model genes were compared between immunotherapy responders and baseline in the GSE109485 and GSE149825 mouse *in vivo* experiments. Responders exhibited higher expression levels of these modeled genes. The baseline group consisted of IgG-treated controls, and the responders were immunotherapy-treated mice that exhibited smaller tumors and longer lifespans *P < 0.05; **P < 0.01; ***P < 0.001. **(D)** Umap plot showing the major cell subtypes in the GSE115978 scRNA-seq immunotherapy dataset. **(E, F)** Expression patterns of the 7 model genes across different cell populations.

To understand the role of model genes in melanoma and immunotherapy response, we first compared the expression levels of seven model genes in tumors and normal tissues. At the transcriptome level, GBP5, TNFSF13B, HLA-DPB1, and CXCL10 had higher expression levels in tumors, whereas CD40, GBP1, and XBP1 were not significantly different (**Supplementary Figures S2A, C, E, G, I, K, L**). These findings were further confirmed by the IHC results from the HPA database (**Supplementary Figures S2B, D, F, H, J**). We then compared the differences in expression levels of model genes between immunotherapy responders and non-responders in 8 additional melanoma immunotherapy cohorts. Consistently, in all 8 immunotherapy cohorts, we observed significantly or modestly higher expression levels of all model genes in responders compared to non-responders (**Figure 7A**).

Given the high homology between human and mouse genes, we used genetically identical mouse melanoma immunotherapy data to further validate the response of the model genes to immunotherapy, thereby avoiding potential interference from differences in the human genome. Similar findings were observed in two *in vivo* mouse studies using a murine melanoma cell line, B16, which underwent anti-cancer immunotherapy (50, 51). The baseline group consisted of IgG-treated controls, and the responders were immunotherapy-treated mice that exhibited smaller tumors and longer lifespans. Compared to the baseline group, all model genes, except Xbp1, showed a trend of significant upregulation in immunotherapy responders (**Figures 7B, C**).

Additionally, we examined the cell-specific expression of model genes using the single-cell RNA-seq (scRNA-seq) dataset of SKCM_GSE115978, an immunotherapy cohort. The UMAP plot revealed the presence of nine distinct cell populations: B cells, CD4^+^ conventional T cells (CD4Tconv), exhausted CD8^+^ T cells (CD8Tex), endothelial cells, fibroblasts, malignant tumor cells, monocytes/macrophages (Mono/Macro), natural killer (NK) cells, and proliferating T cells (Tprolif) (**Figure 7D**). Model genes are predominantly expressed in immune cells such as B cells, CD8Tex, Mono/Macro, NK, and Tprolif (**Figures 7E, F**). Next, we tested whether treatment of ICIs might affect the levels of model genes using scRNA-seq data. Our analysis revealed distinct immune cell type-specific gene expression changes in response to immunotherapy. CD40 expression was up-regulated in Mono/Macro, while GBP5 was up-regulated in B cells, CD8Tex, and Mono/Macro. Similarly, HLA-DPB1 showed upregulation in Mono/Macro, NK, and Tprolif. Other genes, such as CXCL10, GBP1, and TNFSF13B, were also up-regulated in Mono/Macro (**Supplementary Figure S3A**). Together, these results suggest the involvement of model genes like CD40 and GBP5 in the immunotherapy response.

### 3.7 ITRGM is a reliable indicator of the immune landscape

**Figure 8:**
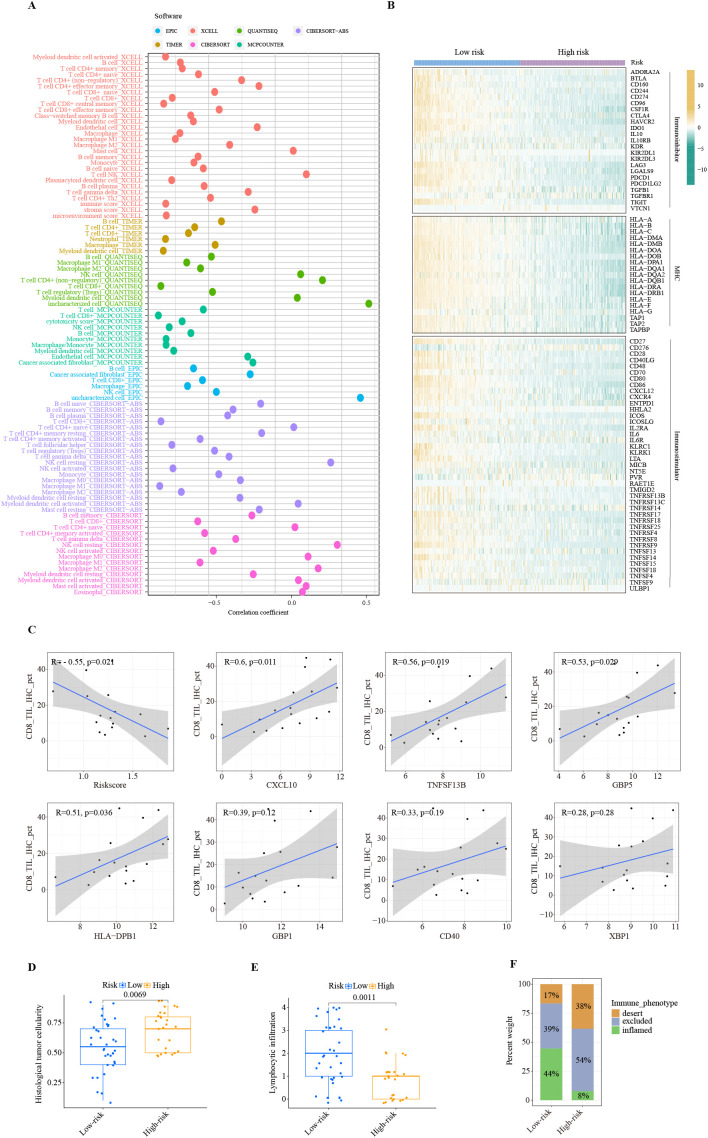
Immune landscape of ITRGM signature. **(A)** Correlation between risk scores based on the ITRGM signature and immune cell infiltration via seven algorithms. Different colors represent different algorithms. **(B)** Heatmap showing the correlation of high and low-risk groups based on ITRGM with immunoinhibitors, MHC, and immunostimulators. **(C)** Correlation of CD8 IHC scores with risk scores based on the ITRGM signature or the expression levels of the seven model genes (CXCL10, TNFSF13B, GBP5, HLA-DPB1, GBP1, CD40, XBP1), respectively in the GSE243238 dataset. The ITRGM signature was negatively associated with CD8 IHC scores, whereas CXCL10, TNFSF13B, GBP5, HLA-DPB1, GBP1, CD40, XBP1 showed positive associations with CD8 IHC scores overall. **(D, E)** Boxplots displaying histological tumor cellularity and lymphocytic infiltration based on histological examination of TCGA-SKCM samples between high and low-risk groups defined by the ITRGM signature. The low-risk group is associated with lower tumor cellularity and higher lymphocytic infiltration. **(F)** Stacked graphs showing the percentage of the desert, excluded, and inflamed immune phenotypes between high and low-risk groups. The low-risk group is associated with a higher percentage of the inflamed immune phenotype.

The TIME strongly influences the efficacy of immunotherapy. Therefore, we conducted a comprehensive investigation of immune-related features associated with risk scores generated by the ITRGM. To investigate the correlation between ITRGM and immune cell infiltration, we employed the ssGSEA algorithm to assess immune cell infiltration in the TCGA-SKCM training set and three additional training sets (GSE22153, GSE54467, GSE69504). Subsequently, based on the ITRGM scores of each dataset, the datasets were divided into high-risk and low-risk groups, and the differences in immune cell infiltration between the groups were explored. The findings indicated that the overall low-risk group exhibited heightened immune cell infiltration in all the TCGA-SKCM training set and the three test sets (**Supplementary Figure S3B**). These results suggested that the ITRGM may differentiate between immune-hot and cold tumors and demonstrated stability. As the TCGA-SKCM cohort serves as the training set and possesses the largest sample size, in addition to accompanying HE pathology slides and tumor mutation data, a more comprehensive analysis utilizing TCGA-SKCM will be conducted subsequently. We further analyzed the levels of immune cell infiltration in the TCGA-SKCM cohort using seven methods. The risk score showed significant negative correlations with many immune cells, most notably CD8^+^ T cells, NK cells, and M1 macrophages (**Figure 8A**; **Supplementary Figure S4A**).

Similarly, the risk score was negatively correlated with the majority of immunoregulators classified as immunoinhibitors, MHC molecules, and immunostimulators (**Figure 8B**). Consistent with this, high infiltration levels of CD8^+^ T cells were significantly associated with superior patient prognosis (**Supplementary Figure S4B**). This was further confirmed by IHC results. The IHC staining scores for CD8 were significantly negatively correlated with the risk score. Importantly, as the risk score is negatively correlated with the levels of model genes, CD8 levels are significantly positively correlated with the model genes CXCL10, TNFSF13B, GBP5, and HLA-DPB1, and positively but not significantly correlated with GBP1, CD40, and XBP1 (**Figure 8C**). These results suggest a strong negative association between the ITRGM risk score and the level of immune cell infiltration within the tumor microenvironment.

Consistently, the H&E staining based on pathology sections also showed that the low-risk group had lower tumor cellularity but higher lymphocyte infiltration (**Figures 8D, E**). Compared with the high-risk group, the low-risk group was dominated by the inflamed-immune phenotype and had a smaller percentage of the desert-immune phenotype (**Figure 8F**). Collectively, these results demonstrate that the risk score generated by the ITRGM is an efficient indicator for TIME, as the risk score is strongly negatively associated with tumor immune infiltration. Therefore, “immune-hot” tumors (low-risk) and “immune-cold” tumors (high-risk) can be reliably distinguished based on the risk score by the ITRGM.

### 3.8 ITRGM is a robust predictive biomarker for immunotherapy

**Figure 9:**
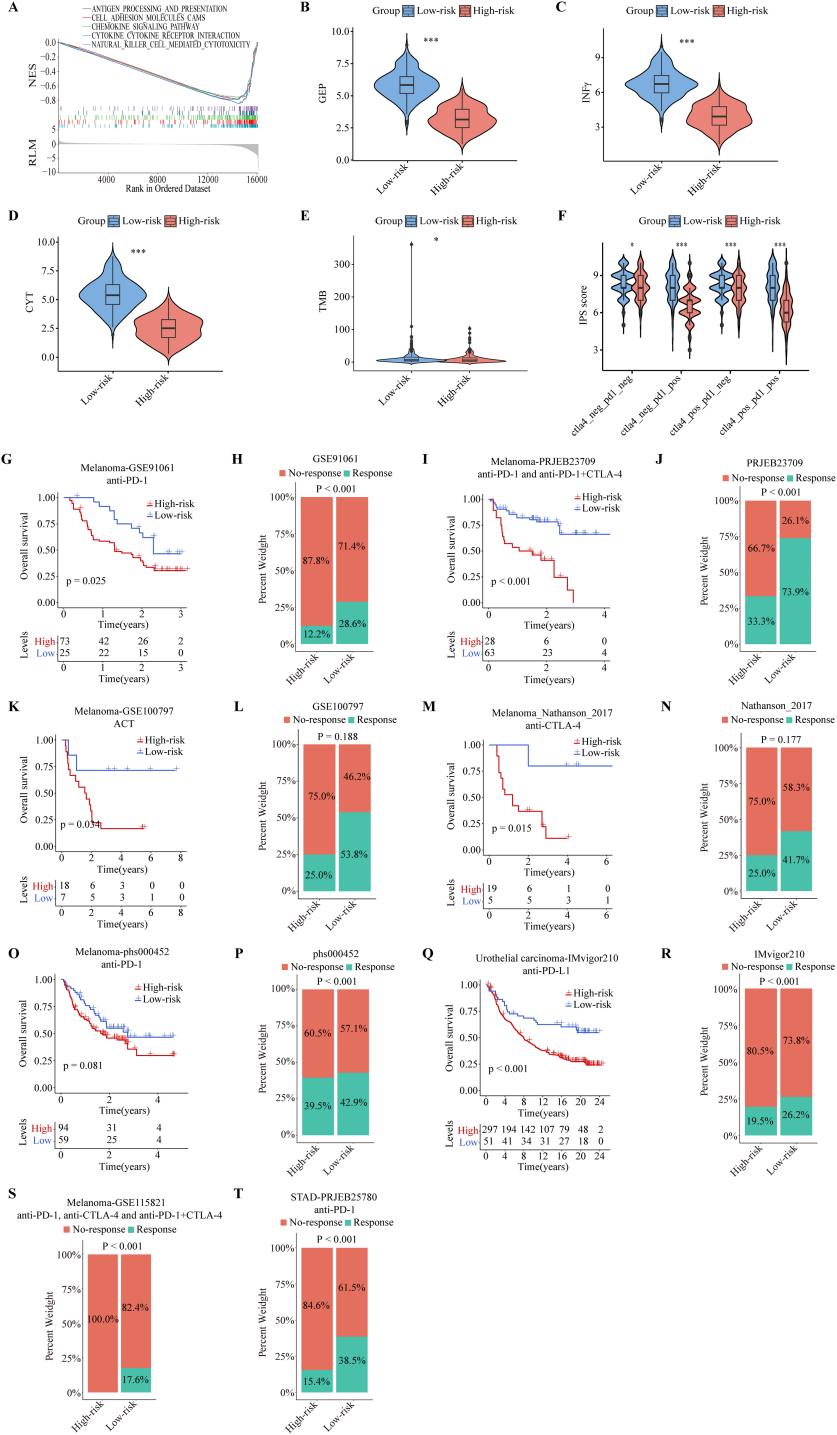
Predictive performance of the ITRGM signature in immunotherapy response. The ITRGM signature stratified each cohort into high-risk or low-risk groups. **(A)** Gene Set Enrichment Analysis (GSEA) between high and low-risk groups in TCGA-SKCM revealed that immune-related pathways were significantly enriched in the low-risk group. **(B-F)** Violin plots display differences in GEP **(B)**, INFγ **(C)**, CYT **(D)**, TMB **(E)**, and IPS scores **(F)** between high and low-risk groups, with the low-risk group demonstrating higher scores across GEP, INFγ, CYT, TMB, and IPS *P < 0.05; ***P < 0.001. Kaplan–Meier survival analyses show OS between high and low-risk groups in the GSE91061 **(G)**, PRJEB23709 **(I)**, GSE100797 **(K)**, Nathanson_2017 **(M)**, phs000452 **(O)**, and IMvigor210, with the low-risk group consistently showing better prognosis. **(Q)**. Stacked graphs show the percentage of immunotherapy responders and non-responders between high and low-risk groups in GSE91061 **(H)**, PRJEB23709 **(J)**, GSE100797 **(L)**, Nathanson_2017 **(N)**, phs000452 **(P)**, IMvigor210 **(R)**, GSE115821 **(S)**, and STAD-PRJEB25780 **(T)**, with the low-risk group showing a higher percentage of immunotherapy responders.

To gain insight into whether ITRGM can be a reliable biomarker for immunotherapy, we performed GSEA analysis of the KEGG gene set between high and low-risk groups defined by ITRGM. Consistent with our prediction, the results showed that many immune regulatory pathways are highly enriched in the low-risk group, such as antigen processing and presentation, chemokine signaling pathway, and natural killer cell-mediated cytotoxicity (**Figure 9A**). Next, we tested whether the ITRGM signature is related to several commonly used immunotherapy predictors. Remarkably, the immune-related gene expression profile (GEP), interferon gamma (INFγ), cytolytic activity (CYT), and TMB displayed significantly higher values in the low-risk group compared to the high-risk group, suggesting potentially more favorable immunotherapy outcomes in the low-risk group (**Figures 9B–E**). Additionally, we found that the low-risk group exhibited higher immunophenoscore (IPS) values, a feature positively linked with immunotherapy, suggesting better prognosis for immunotherapy response (**Figure 9F**).

To further evaluate the efficacy of the ITRGM signature in predicting immunotherapy outcomes, we included eight immunotherapy cohorts for further validation analysis, including six melanoma immunotherapy cohorts, one urothelial carcinoma immunotherapy cohort, and one stomach adenocarcinoma (STAD) immunotherapy cohort. We applied ITRGM to these 8 immunotherapy cohorts. Consistently, Kaplan-Meier analysis showed that the low-risk group had a superior prognosis in 5 melanoma immunotherapy cohorts (GSE91061, PRJEB23709, GSE100797, Nathanson-2017, phs000452) with survival data (**Figures 9G, I, K, M, O**), and similarly in the IMvigor210 immunotherapy cohort (**Figure 9Q**). Stacked plots showed that the low-risk group had higher proportions of immunotherapy responders in all eight immunotherapy cohorts (**Figures 9H, J, L, N, P, R–T**). Our results provide convincing evidence that patients with a low ITRGM signature, or low risk, benefit more from immunotherapy than patients with a high ITRGM signature, demonstrating that ITRGM is a promising predictive biomarker for immunotherapy response.

### 3.9 GBP5 expression correlates with CD8^+^ T cell infiltration

**Figure 10:**
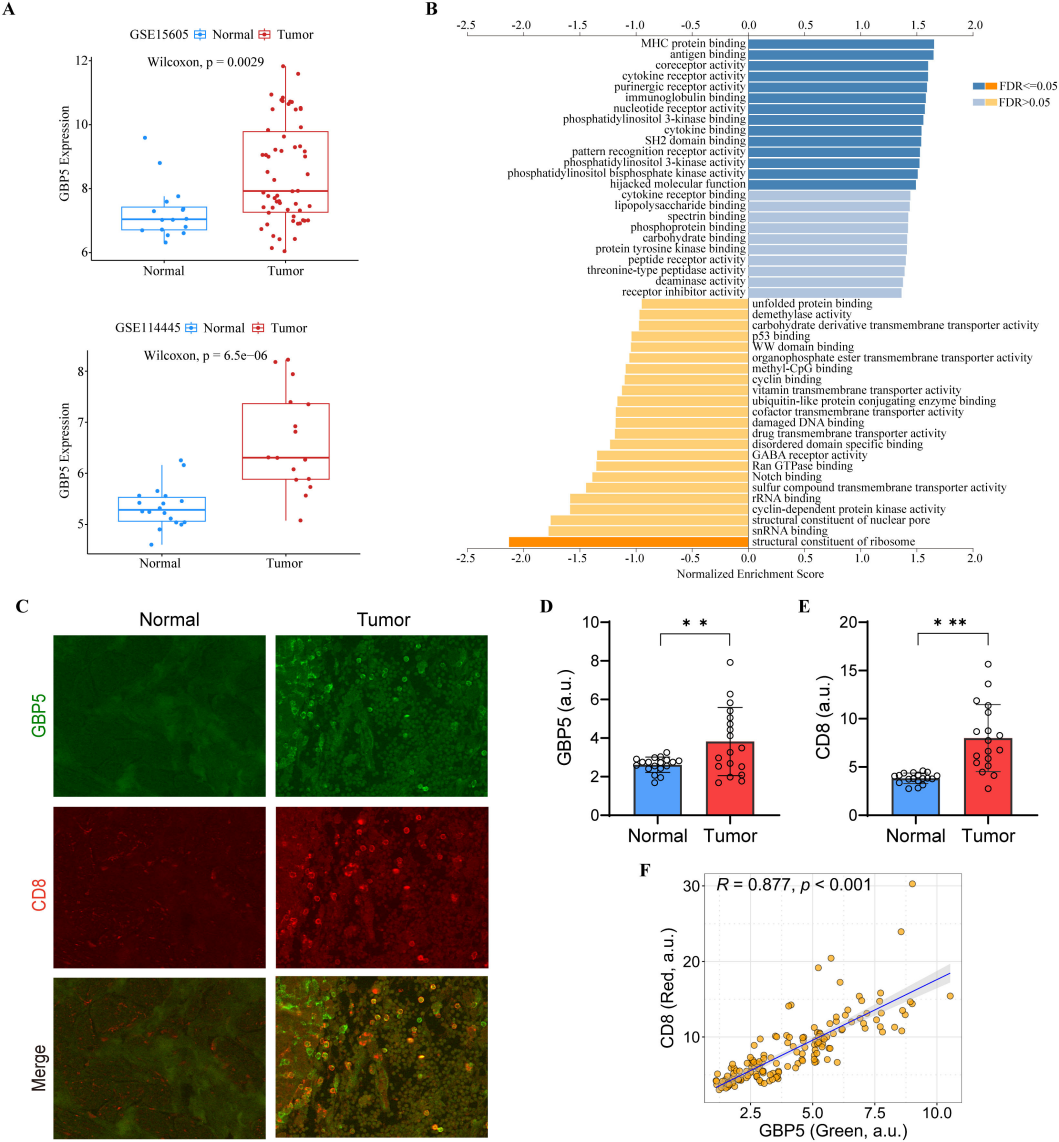
The Role of GBP5 in melanoma. **(A)** The expression levels of GBP5 in tumor tissues compared to normal tissues in the GSE15605 (top) and GSE114445 (bottom) melanoma datasets, with tumor tissues exhibiting higher GBP5 expression levels. **(B)** Gene Set Enrichment Analysis (GSEA) of GBP5 using LinkedOmics. **(C)** Representative immunostaining images of GBP5 and CD8 in the melanoma tissues and normal skin tissues reveal that tumor tissue exhibit higher levels of GBP5 and CD8 signatures. Scale bars, 50 μm. **(D, E)** Bar graphs show relative expression levels of GBP5 **(D)** and CD8 **(E)** in melanoma tissues (N = 17) and normal skin tissue (N = 18). Tumor tissues are associated with higher levels of GBP5 and CD8. **P < 0.01; ***P < 0.001. **(F)** Analysis of correlation between GBP5 and CD8 expression within tumor tissues revealed a positive relationship between the two markers. Each dot represents a ROI and 3-10 ROIs were selected from each tumor section (N = 156). a.u., arbitrary unit.

Notably, all seven model genes have been shown to play diverse roles in immunity and immunotherapy (52–66). In particular, GBP5 has emerged as an immune regulator and a biomarker for inflammation and cancers (67–69). GBP5 promotes the assembly of the NLRP3-containing inflammasome and activates the NF-κB signaling pathway (69–71). It has also been linked to the immune microenvironment, where it plays a role in influencing tumor progression (68). To further elucidate the role of GBP5 in the ITRGM signature, we conducted additional bioinformatics analysis on GBP5. In addition to the TCGA-SKCM cohort (**Supplementary Figure S2A**), two additional melanoma cohorts (GSE15605, GSE114445) were included. Consistently, GBP5 showed higher expression levels in tumor tissues relative to normal tissues (**Figure 10A**). GSEA analysis revealed that GBP5 exhibited a significant positive correlation with numerous immune-related pathways, such as MHC protein binding, antigen binding, and immunoglobulin binding (**Figure 10B**). Analysis of immune cell infiltration revealed a positive correlation between GBP5 and various immune cells, including macrophage M1, NK cells, B cells, and CD8^+^ T cells, with CD8^+^ T cells showing the strongest correlation (**Supplementary Figure S5A**). Additionally, the association of GBP5 with CD8^+^ T cell infiltration was investigated across 33 cancers. Except for thymoma (THYM), GBP5 was significantly and positively associated with CD8^+^ T cell infiltration in 32 other cancers (**Supplementary Figure S5B**). Finally, through our IHC experiments using tissue microarrays containing 17 cases of melanoma and 18 cases of normal skin tissues, we confirmed that GBP5 exhibited significantly higher expression levels in melanoma tumor tissues compared to normal tissues (**Figures 10C, D**). Similarly, higher infiltration levels of CD8^+^ T cells were observed in tumor tissues compared to normal tissues (**Figures 10C, E**). Importantly, the expression levels of GBP5 showed a significant positive correlation with CD8^+^ T cell infiltration levels (**Figure 10F**), corroborating our findings that ITRGM, which includes seven model genes, notably GBP5, is a promising biomarker for immunotherapy in melanoma.

### 3.10 Evaluation of ITRGM signature in chemotherapy efficacy

In clinical practice, immunotherapy is rarely used alone; it is often combined with chemotherapy to achieve better outcomes. To further explore the clinical implications of ITRGM, we assessed the therapeutic efficacy of various chemotherapeutic agents across low and high-risk groups using the oncopredict package (47). The low-risk group had a better response to temozolomide and cisplatin, both of which are commonly used for chemotherapy to treat melanoma (**Supplementary Figure S5C**). Similarly, higher risk scores indicated higher resistance to these chemotherapy drugs (**Supplementary Figure S5D**). Interestingly, the high-risk group showed a lower resistance to sorafenib and afatinib. (**Supplementary Figure S5E**), with a negative correlation with the risk score (**Supplementary Figure S5F**). Sorafenib, a multikinase inhibitor, has shown potential to treat melanoma (72, 73). Afatinib, a tyrosine kinase inhibitor used for the treatment of non-small cell lung cancer, has also shown therapeutic potential for melanoma (74, 75). This analysis suggests that temozolomide and cisplatin may enhance the sensitivity of immunotherapy for the low-risk group, while sorafenib and afatinib may provide beneficial outcomes for the high-risk group in combination with immunotherapy.

## 4 Discussion

The incidence and morbidity of SKCM have increased in successive decades (2, 76). Despite advancements in therapies such as surgery, chemotherapy, targeted therapies, and radiotherapy, their impact on patients with malignant melanoma has been limited (10). Immunotherapy, however, has revolutionized melanoma treatment and significantly enhanced patient survival with landmark approvals (77). Yet, the high cost and variable patient responses often lead to challenges of over- or under-treatment (78). Therefore, establishing a reliable biomarker is crucial to identify SKCM patients who are likely to benefit most from immunotherapy.

This study aims to develop a stable and robust signature based on immunotherapy-related genes using a novel computational framework, and to explore its implications from multiple perspectives. Firstly, we identified a consensus of 66 genes associated with immunotherapy, termed CITPGs, through WGCNA and DEG analysis. These genes are pivotal in immune function, impact patient prognosis, and exhibit a high mutation rate in melanoma, underscoring their potential role in disease progression. Secondly, based on these CITPGs, SKCM can be classified into two distinct subtypes. One subtype comprises “immune-hot” tumors characterized by higher immune infiltration levels, better prognosis, and greater responsiveness to immunotherapy. In contrast, the other subtype includes “immune-cold” tumors with lower immune infiltration, poorer prognosis, and reduced response rates to immunotherapy.

Subsequently, we screened for DEGs between CITPG-high and CITPG-low groups across four melanoma cohorts (TCGA-SKCM, GSE22153, GSE54467, GSE69504), identifying a set of 44 common DEGs. To construct an optimal predictive model, we evaluated these 44 genes using 101 machine learning algorithms, selecting “Lasso+plsRcox” as the best-performing model. The resulting seven-gene ITRGM signature demonstrated superior predictive power compared to traditional clinical indicators such as gender, age, and stage. When compared with 37 previously published signatures across multiple datasets (TCGA-SKCM training, 3 GSE cohorts, and a meta-cohort), ITRGM consistently showed higher predictive accuracy based on C-index assessments (**Figure 6C**). Notably, while signatures like Li_G, Zhong_L, and Cao_X performed comparably well in certain cohorts, their performance varied significantly across different datasets, indicating limited generalizability likely due to overfitting. In contrast, our ITRGM signature, optimized through dimensionality reduction and machine learning, demonstrated enhanced stability and potential for broader applicability across diverse melanoma patient cohorts.

The TIME, a complex regulator of cancer progression, is a central focus of immunotherapy (79, 80). Melanoma patients classified with a low-risk score based on the ITRGM signature exhibit extensive infiltration of immune cells, notably CD4^+^ T cells, M1-polarized macrophages, CD8^+^ T cells, NK cells, and B cells. These immune cell populations are associated with improved prognosis and extended survival in patients undergoing immunotherapy (79, 81, 82), as corroborated by CD8 immunohistochemistry and H&E pathology sections aligning with transcriptomic assessments. Furthermore, patients with low-risk scores based on the ITRGM signature demonstrate heightened activity in immune-related signaling pathways, including GEP, IFNγ, CYT, TMB, and IPS scores, all of which are indicators of favorable responses to immunotherapy. These findings suggest that melanoma patients identified as low-risk by the ITRGM signature are more likely to benefit from immunotherapy. To validate this hypothesis, the ITRGM signature was evaluated across six melanoma immunotherapy cohorts (GSE91061, PRJEB23709, GSE100797, Nathanson-2017, phs000452, and GSE115821), one stomach adenocarcinoma cohort (PRJEB25780), and one urothelial carcinoma cohort (IMvigor210). Consistently, patients classified with a low-risk score by ITRGM exhibited superior responses to immunotherapy and better overall prognosis across all these immunotherapy cohorts (**Figure 9**). These results underscore the potential clinical utility of the ITRGM signature in guiding the management of melanoma patients, and potentially other cancers, treated with immunotherapy.

The ITRGM signature includes GBP5, HLA-DPB1, XBP1, CD40, CXCL10, and TNFSF13B, each playing pivotal roles in the immune response and tumor microenvironment of melanoma. GBP5, a member of the TRAFAC class dynamin-like GTPase superfamily, is involved in inflammasome assembly and innate immunity (83, 84), and promotes M1 macrophage polarization, suggesting potential roles in enhancing anti-tumor immunity (85, 86). HLA-DPB1, critical for presenting extracellular peptides, shows reduced expression in melanoma patients resistant to immune checkpoint therapy, indicating its relevance as a prognostic marker (61). XBP1, a transcription factor regulating MHC class II expression, has dual roles in melanoma—potentially enhancing anti-tumor immunity through dendritic cells and NK cells (87–89), while also exhibiting immunosuppressive properties in melanoma (65, 90). CD40, a member of the TNF receptor superfamily, enhances T-cell activity and has shown promise in clinical trials with CD40 agonistic antibodies combined with immune checkpoint inhibitors for treating metastatic melanoma (52, 54, 91, 92). CXCL10, upregulated in melanoma tissues (93), recruits CD8^+^ T cells and NK cells to tumors (55, 94), promoting anti-tumor immune responses (55, 57, 94), and serving as a predictive marker for immunotherapy outcomes (95). TNFSF13B (APRIL), a proinflammatory cytokine, supports T-cell survival and enhances dendritic cell functions in melanoma and other cancers (63). These genes collectively influence the TIME and hold substantial implications for immunotherapy response and patient prognosis in melanoma. They underscore the ITRGM signature as a robust tool with promising potential to accurately predict treatment outcomes and guide personalized therapy decisions for melanoma patients undergoing immunotherapy.

Despite its promise for predicting immunotherapy response in melanoma, ITRGM has limitations to consider for clinical translation. First, all datasets originated from single-center retrospective studies. Validation in prospective multicenter cohorts with larger and more diverse patient populations is crucial to confirm ITRGM’s generalizability and effectiveness in real-world settings. Second, the seven genes in ITRGM, known for their roles in immune response and immunotherapy, warrant further investigation for their performance as biomarkers in other cancers. Our findings in **Figure 9** suggest that ITRGM may have broad applicability, but studies focusing on other tumor types are needed to determine its accuracy and clinical utility across different cancers.

## 5 Conclusion

In conclusion, we developed a robust 7-gene signature, ITRGM, for predicting prognosis and immunotherapy response in melanoma patients. This signature integrates data from bulk RNA-seq, scRNA-seq, pathology, and IHC via multiple bioinformatics analyses and 101 machine learning combinatorial algorithms. Notably, ITRGM accurately stratifies prognosis in other cancers like urothelial carcinoma and STAD, suggesting broad applicability across multiple tumor types. Overall, our findings support ITRGM as a promising tool for enhancing personalized treatment and improving clinical management of melanoma and potentially other cancers.

## Data Availability

The datasets presented in this study can be found in online repositories. The names of the repository/repositories and accession number(s) can be found in the article/**Supplementary Material**.

## Supplementary material

Supplementary Figure 1 Consensus clustering analysis and mutation frequency analysis of 66 CITPGs in TCGA-SKCM. **(A)** Soft-threshold power determination. The top panel displays the scale-free fit index on the y-axis, indicating how well the network conforms to a scale-free topology. The bottom panel shows the mean connectivity on the y-axis, representing the average degree of connectivity within the network. The x-axis indicates the power value of the soft threshold, which is used to adjust the network’s scale-free topology. **(B)** The dendrogram illustrates gene clustering based on the dissimilarity metric (1-TOM), showing that 18 gene modules were established. The y-axis on the left, labeled as “Height,” represents the dissimilarity or distance between gene expression profiles, with lower values indicating more similar gene expressions. The dendrogram branches depict the hierarchical clustering of genes based on their expression profiles. The x-axis lists individual genes from the patient samples, representing all the genes analyzed. Below the dendrogram, a colored horizontal bar labeled “Dynamic Tree Cut” shows the results of dynamic tree cutting, a method used to define gene modules. Each module is represented by a distinct color, and all genes within a module share the same color. These modules are groups of genes that exhibit highly correlated expression patterns, suggesting that they may be co-regulated or functionally related. **(C)** Mutation frequency analysis of the 66 CITPGs in TCGA-SKCM cohort. **(D)** Consensus cumulative distribution function (CDF) in TCGA-SKCM, PRJEB23709, and GSE91061 datasets. **(E)** Delta area in TCGA-SKCM, PRJEB23709, and GSE91061 datasets. **(F)** PCA analysis for two clusters based on CITPGs-expression in TCGA-SKCM, PRJEB23709, and GSE91061 datasets.

Supplementary Figure 2 Box plots and IHC images of the 7 model genes. Box plots show the mRNA levels of GBP5 **(A)**, TNFSF13B **(C)**, HLA-DPB1 **(E)**, CD40 **(G)**, GBP1 **(I)**, CXCL10 **(K)**, and XBP1 **(L)** between melanoma tissues (red bar) and normal tissues (grey bar) from the GEPIA2 database. Representative IHC images of GBP5 **(B)**, TNFSF13B **(D)**, HLA-DPB1 **(F)**, CD40 **(H)**, and GBP1 **(J)** from HPA database.

Supplementary Figure 3 Implication of ITRGM in immunotherapy responses. **(A)** The expression levels of the 7 model genes were analyzed with and without immunotherapy treatment across various cell populations using a scRNA-seq dataset of SKCM_GSE115978. **(B)** Histograms show the level of immune cell infiltration between high-risk (red) and low-risk groups (green) in the GSE22153, GSE54467, GSE65904, and TCGA-SKCM cohorts, showing the low-risk group being associated with higher immune cell infiltration level.

Supplementary Figure 4 Association of CD8+ T cells with the ITRGM signature and prognosis in SKCM patients. **(A)** The correlation of the ITRGM signature with CD8^+^ T cells was assessed by multiple algorithms in the TCGA-SCKM cohort, revealing a negative correlation between the ITRGM signature and CD8+ T cells. **(B)** Kaplan-Meier survival analysis was performed based on CD8^+^ T cell infiltration levels in TCGA-SKCM patients revealed that higher CD8+ T-cell infiltration is associated with a better prognosis.

Supplementary Figure 5 Immune infiltration analysis and drug sensitivity analysis. **(A)** Correlation between GBP5 expression level and immune cell infiltration was assessed with multiple algorithms. CD8^+^ T cells are marked with red boxes in the TCGA-SCKM cohort. **(B)** Correlation between GBP5 expression level and CD8^+^ T cell infiltration in 32 cancer types from TCGA was assessed with multiple algorithms, revealing a positive correlation between GBP5 and CD8+ T cell infiltration. **(C, E)** The chemotherapy sensitivity estimation of 4 drugs (Temozolomide, Cisplatin, Sorafenib, Afatinib) was estimated between the high-risk and low-risk groups in the TCGA-SCKM cohort. The low-risk group exhibited greater sensitive to Temozolomide and Cisplatin but showed higher resistant to Sorafenib and Afatinib. **(D, F)** Correlation analysis was performed to assess the relationship between chemotherapy sensitivity of 4 drugs (Temozolomide, Cisplatin, Sorafenib, Afatinib) and risk scores in the TCGA-SCKM cohort. Risk score were positively associated with resistance to Temozolomide and Cisplatin, but negatively associated with resistance to Sorafenib and Afatinib.
