# Supplementary figures and images for: Machine learning-based identification of an immunotherapy-related signature to enhance outcomes and immunotherapy responses in melanoma
Source: Front Immunol. 2024 Sep 17;15:1451103. doi: 10.3389/fimmu.2024.1451103 (PMC11442245; doi:10.3389/fimmu.2024.1451103)

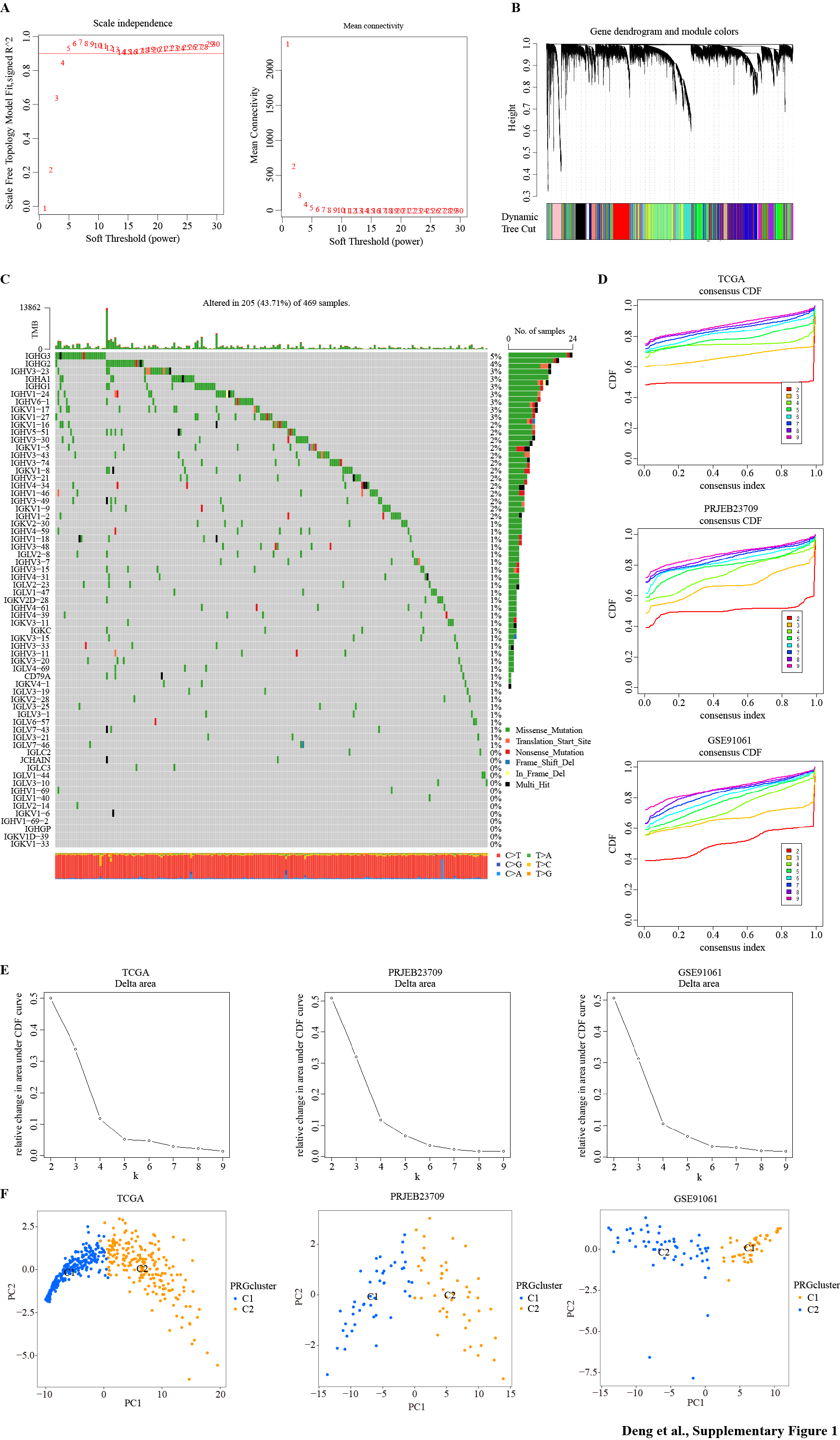

Supplement: Supplementary Figure 1 — Consensus clustering analysis and mutation frequency analysis of 66 CITPGs in TCGA-SKCM. (A) Soft-threshold power determination. The top panel displays the scale-free fit index on the y-axis, indicating how well the network conforms to a scale-free topology. The bottom panel shows the mean connectivity on the y-axis, representing the average degree of connectivity within the network. The x-axis indicates the power value of the soft threshold, which is used to adjust the network’s scale-free topology. (B) The dendrogram illustrates gene clustering based on the dissimilarity metric (1-TOM), showing that 18 gene modules were established. The y-axis on the left, labeled as “Height,” represents the dissimilarity or distance between gene expression profiles, with lower values indicating more similar gene expressions. The dendrogram branches depict the hierarchical clustering of genes based on their expression profiles. The x-axis lists individual genes from the patient samples, representing all the genes analyzed. Below the dendrogram, a colored horizontal bar labeled “Dynamic Tree Cut” shows the results of dynamic tree cutting, a method used to define gene modules. Each module is represented by a distinct color, and all genes within a module share the same color. These modules are groups of genes that exhibit highly correlated expression patterns, suggesting that they may be co-regulated or functionally related. (C) Mutation frequency analysis of the 66 CITPGs in TCGA-SKCM cohort. (D) Consensus cumulative distribution function (CDF) in TCGA-SKCM, PRJEB23709, and GSE91061 datasets. (E) Delta area in TCGA-SKCM, PRJEB23709, and GSE91061 datasets. (F) PCA analysis for two clusters based on CITPGs-expression in TCGA-SKCM, PRJEB23709, and GSE91061 datasets. [file Image1.tif]

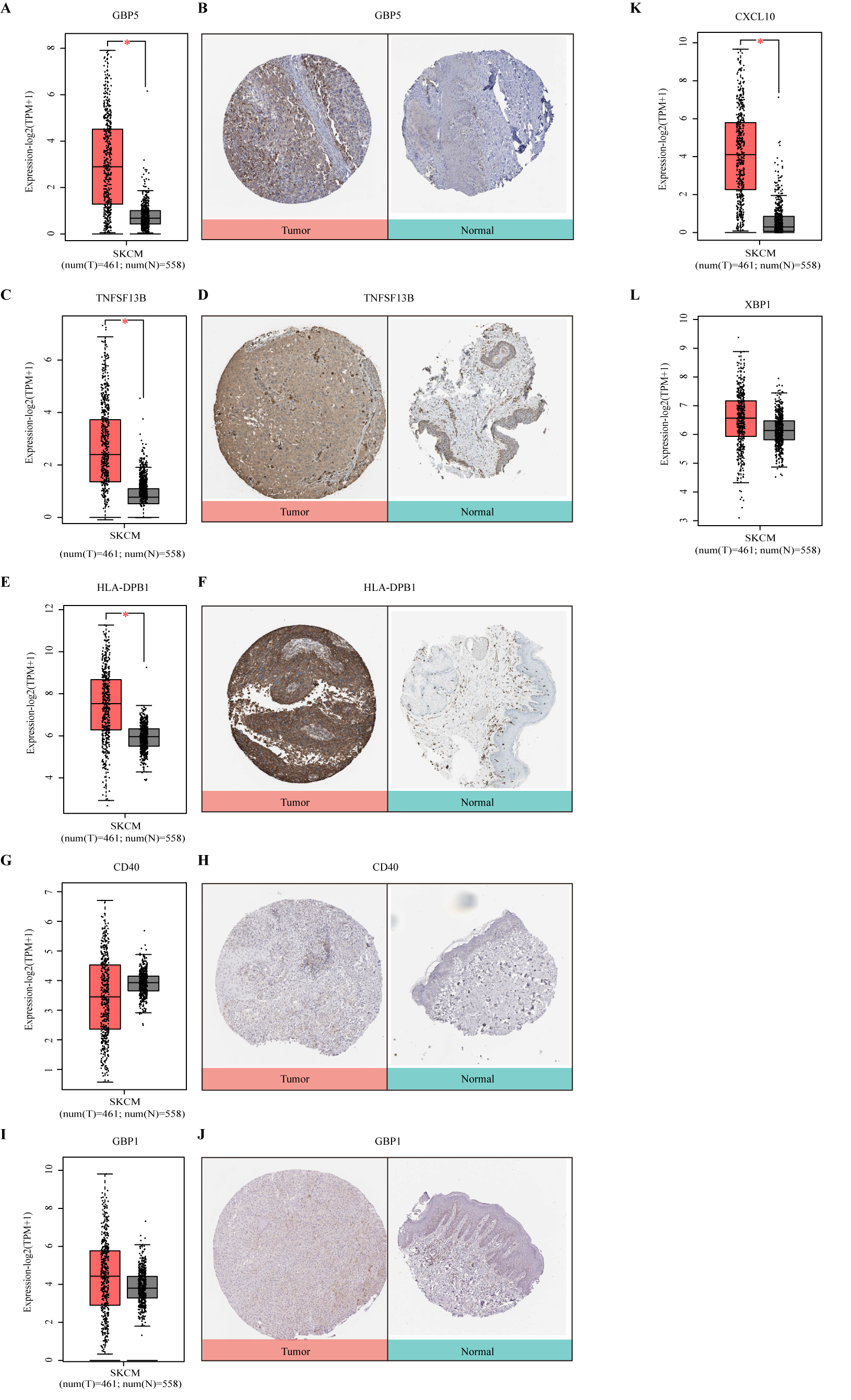

Supplement: Supplementary Figure 2 — Box plots and IHC images of the 7 model genes. Box plots show the mRNA levels of GBP5 (A), TNFSF13B (C), HLA-DPB1 (E), CD40 (G), GBP1 (I), CXCL10 (K), and XBP1 (L) between melanoma tissues (red bar) and normal tissues (grey bar) from the GEPIA2 database. Representative IHC images of GBP5 (B), TNFSF13B (D), HLA-DPB1 (F), CD40 (H), and GBP1 (J) from HPA database. [file Image2.tif]

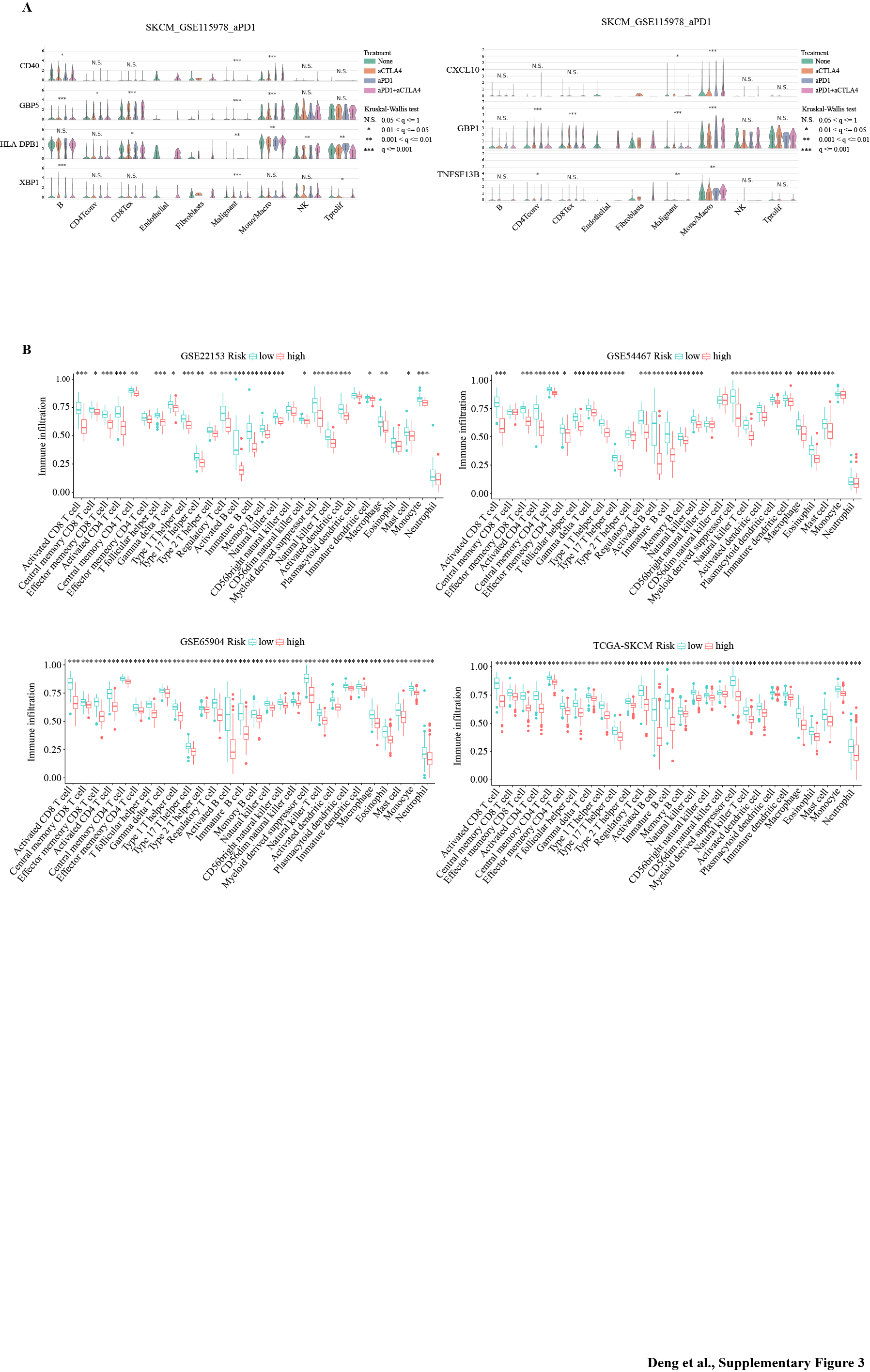

Supplement: Supplementary Figure 3 — Implication of ITRGM in immunotherapy responses. (A) The expression levels of the 7 model genes were analyzed with and without immunotherapy treatment across various cell populations using a scRNA-seq dataset of SKCM_GSE115978. (B) Histograms show the level of immune cell infiltration between high-risk (red) and low-risk groups (green) in the GSE22153, GSE54467, GSE65904, and TCGA-SKCM cohorts, showing the low-risk group being associated with higher immune cell infiltration level. [file Image3.tif]

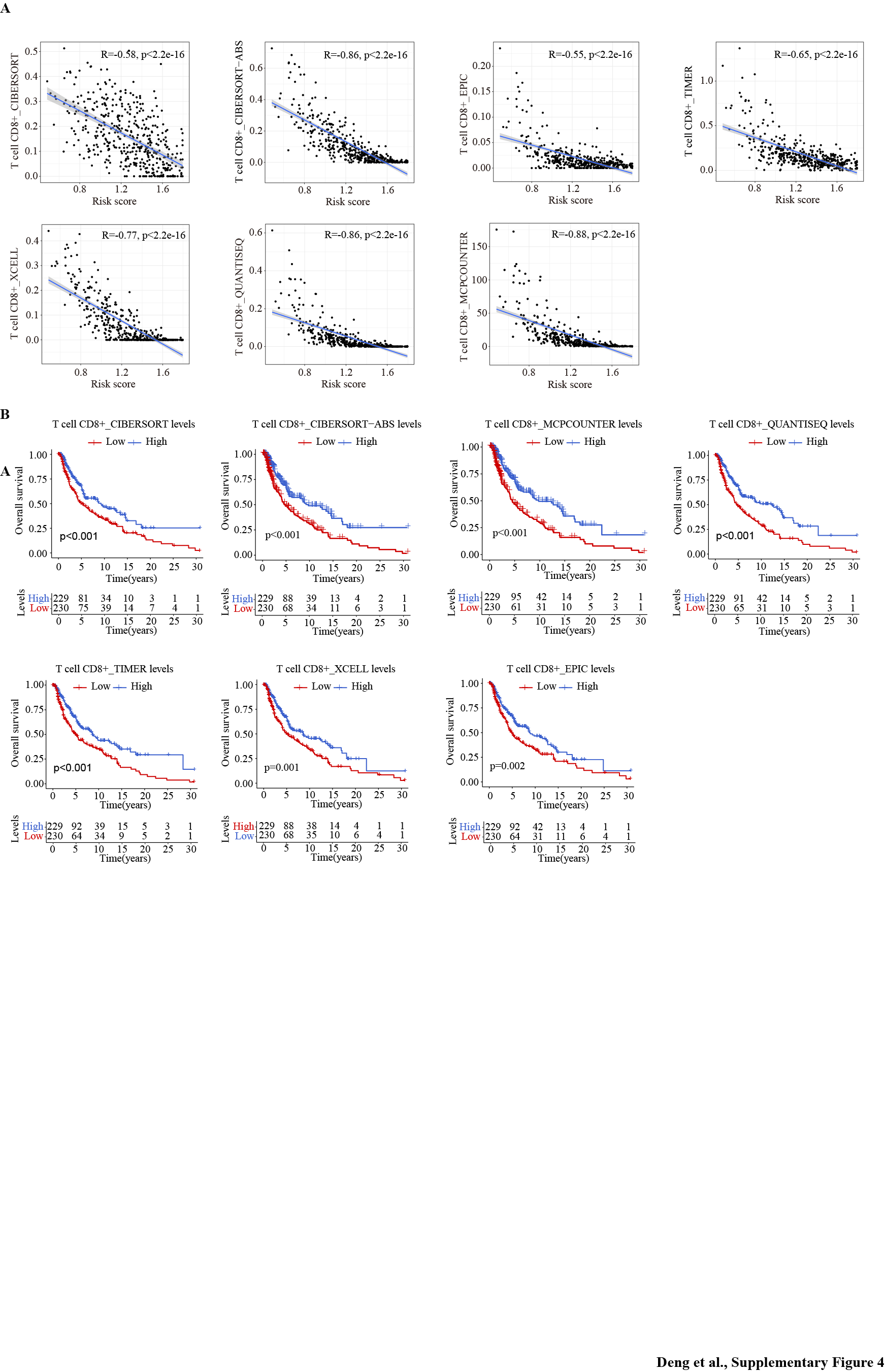

Supplement: Supplementary Figure 4 — Association of CD8+ T cells with the ITRGM signature and prognosis in SKCM patients. (A) The correlation of the ITRGM signature with CD8+ T cells was assessed by multiple algorithms in the TCGA-SCKM cohort, revealing a negative correlation between the ITRGM signature and CD8+ T cells. (B) Kaplan-Meier survival analysis was performed based on CD8+ T cell infiltration levels in TCGA-SKCM patients revealed that higher CD8+ T-cell infiltration is associated with a better prognosis. [file Image4.tif]

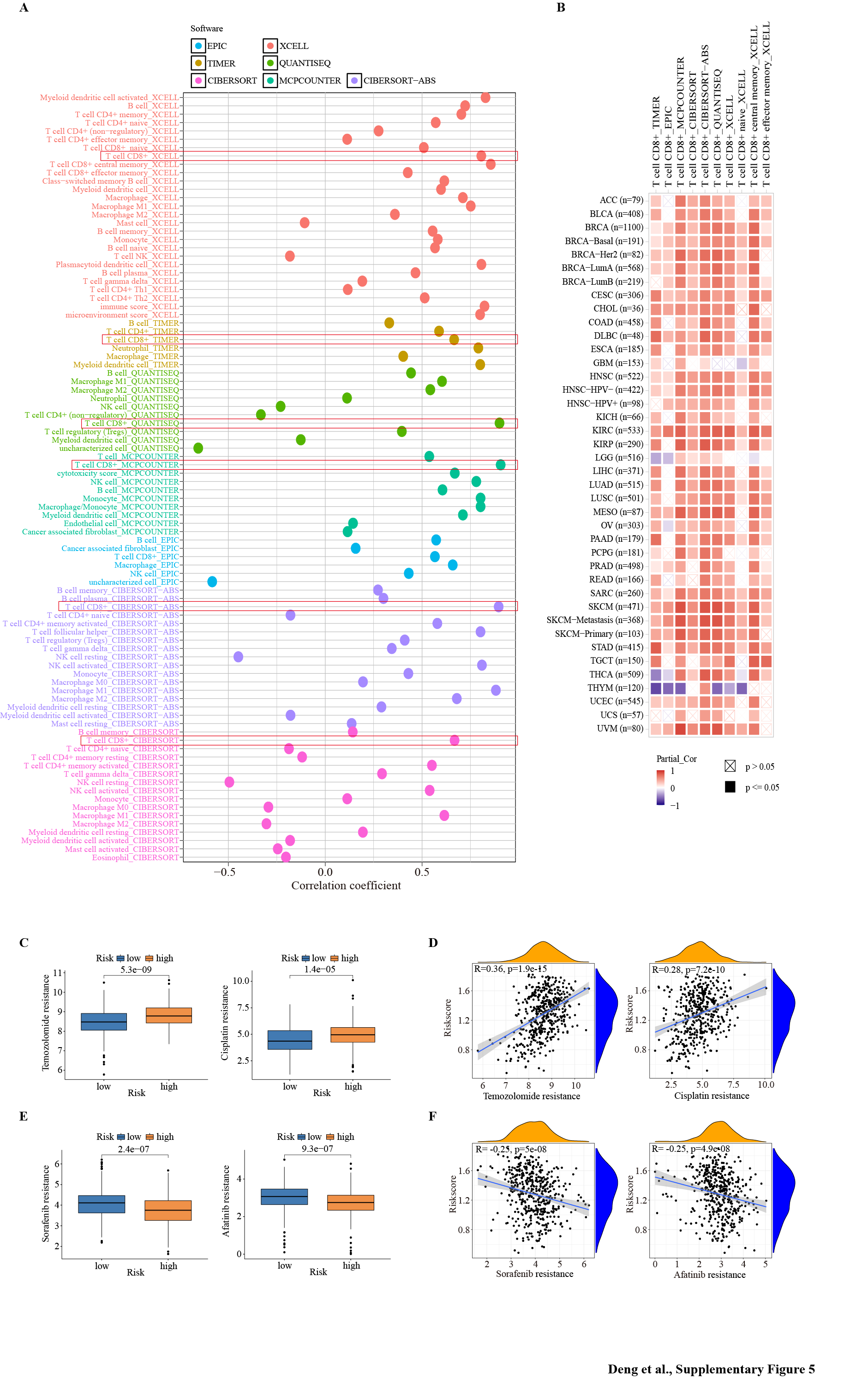

Supplement: Supplementary Figure 5 — Immune infiltration analysis and drug sensitivity analysis. (A) Correlation between GBP5 expression level and immune cell infiltration was assessed with multiple algorithms. CD8+ T cells are marked with red boxes in the TCGA-SCKM cohort. (B) Correlation between GBP5 expression level and CD8+ T cell infiltration in 32 cancer types from TCGA was assessed with multiple algorithms, revealing a positive correlation between GBP5 and CD8+ T cell infiltration. (C, E) The chemotherapy sensitivity estimation of 4 drugs (Temozolomide, Cisplatin, Sorafenib, Afatinib) was estimated between the high-risk and low-risk groups in the TCGA-SCKM cohort. The low-risk group exhibited greater sensitive to Temozolomide and Cisplatin but showed higher resistant to Sorafenib and Afatinib. (D, F) Correlation analysis was performed to assess the relationship between chemotherapy sensitivity of 4 drugs (Temozolomide, Cisplatin, Sorafenib, Afatinib) and risk scores in the TCGA-SCKM cohort. Risk score were positively associated with resistance to Temozolomide and Cisplatin, but negatively associated with resistance to Sorafenib and Afatinib. [file Image5.tif]
