# Supplementary material for: Machine learning-based identification of an immunotherapy-related signature to enhance outcomes and immunotherapy responses in melanoma
Source: Front Immunol. 2024 Sep 17;15:1451103. doi: 10.3389/fimmu.2024.1451103 (PMC11442245; doi:10.3389/fimmu.2024.1451103)
Supplement: Supplementary file 8 [file Table1.docx]

| Dataset type | Data sets | PMID | Source | Cancer Type | Number of samples | Survival time (days) | Therapy | # Responder | # No-Responder |
| --- | --- | --- | --- | --- | --- | --- | --- | --- | --- |
| Melanoma cohorts | TCGA-SKCM |  | GDC | Melanoma | 459 | 0~11252 |  |  |  |
|  | GSE54467 | 24975271 | GEO | Melanoma | 79 | 121~12094 |  |  |  |
|  | GSE65904 | 25909218 | GEO | Melanoma | 210 | 6~6453 |  |  |  |
|  | GSE22153 | 20460471 | GEO | Melanoma | 54 | 10~3509 |  |  |  |
|  | GSE243238 | 37857525 | GEO | Melanoma | 17 |  |  |  |  |
|  | GSE15605 | 23633021 | GEO | Melanoma | 74 |  |  |  |  |
|  | GSE114445 | 30326165 | GEO | Melanoma | 34 |  |  |  |  |
| Immunotherapy cohort | GSE100797 | 29170503 | TIGER | Melanoma | 25 | 96~2787 | ACT | 10 | 15 |
|  | GSE91061 | 29033130 | TIGER | Melanoma | 98 | 57~1143 | anti-PD-1 | 20 | 78 |
|  | phs000452 | 26359337 | TIGER | Melanoma | 153 | 40~1691 | anti-PD-1 | 63 | 90 |
|  | IMvigor210 | 26952546 | TIGER | Urothelial carcinoma | 348 | 72~8934 | anti-PD-L1 | 68 | 230 |
|  | PRJEB23709 | 30753825 | TIGER | Melanoma | 91 | 22~1603 | anti-PD-1, anti-CTLA-4 + anti-PD-1 | 49 | 42 |
|  | Nathanson_2017 | 27956380 | TIGER | Melanoma | 24 | 146~2518.5 | anti-CTLA-4 | 8 | 16 |
|  | GSE115821 | 30127394 | TIGER | Melanoma | 37 |  | anti-CTLA-4, anti-PD-1, anti-CTLA-4 + anti-PD-1 | 3 | 34 |
|  | GSE135222 | 31537801 | TIGER | NSCLC | 27 |  | anti-PD-1 | 8 | 19 |
|  | PRJEB25780 | 30013197 | TIGER | STAD | 78 |  | anti-PD-1 | 21 | 57 |
| scRNA seq cohort | GSE115978 | 30388455 | TIMER2 | Melanoma |  |  | anti-CTLA-4, anti-PD-1, anti-CTLA-4 + anti-PD-1 |  |  |

**Table 1**. List of cohorts used in this study.

GDC, Genomic Data Commons; GEO, Gene Expression Omnibus; TIGER, Tumor Immunotherapy Gene Expression Resource; NSCLC, non-small-cell lung cancer; STAD, stomach adenocarcinoma; ACT, Adoptive Cell Transfer
